# Advances in the study of cancer metastasis and calcium signaling as potential therapeutic targets

**DOI:** 10.37349/etat.2021.00046

**Published:** 2021-06-28

**Authors:** Chaochu Cui, Yongxi Zhang, Gang Liu, Shuhong Zhang, Jinghang Zhang, Xianwei Wang

**Affiliations:** 1 Henan Key Laboratory of Medical Tissue Regeneration, College of Basic Medical Sciences, Xinxiang Medical University, Xinxiang 453003, Henan, China; 2 Department of Oncology, The Third Affiliated Hospital of Xinxiang Medical University, Xinxiang 453003, Henan, China; 3 Department of Pathology, The First Affiliated Hospital of Xinxiang Medical University, Xinxiang 453003, Henan, China; The University of Texas at Arlington, USA

**Keywords:** Calcium channel, cancer metastasis, epithelial-mesenchymal transition, tumor microenvironment, immunosurveillance, metastatic colonization

## Abstract

Metastasis is still the primary cause of cancer-related mortality. However, the underlying mechanisms of cancer metastasis are not yet fully understood. Currently, the epithelial-mesenchymal transition, metabolic remodeling, cancer cell intercommunication and the tumor microenvironment including diverse stromal cells, are reported to affect the metastatic process of cancer cells. Calcium ions (Ca^2+^) are ubiquitous second messengers that manipulate cancer metastasis by affecting signaling pathways. Diverse transporter/pump/channel-mediated Ca^2+^ currents form Ca^2+^ oscillations that can be decoded by Ca^2+^-binding proteins, which are promising prognostic biomarkers and therapeutic targets of cancer metastasis. This paper presents a review of the advances in research on the mechanisms underlying cancer metastasis and the roles of Ca^2+^-related signals in these events.

## Introduction

Cancer metastasis is a process in which cancer cells spread from the primary site to secondary sites. Cancer metastasis is affected by chemoattractants [1], the tumor microenvironment (TME) [2], tissue topography [3], the epithelial-mesenchymal transition (EMT), metabolic remodeling [4, 5], communication between cancer cells [6], etc. Despite the significant achievements in the diagnosis of cancer and therapy interventions, cancer-related death remains an urgent problem worldwide, with cancer metastasis being the major challenge [7–9]. Firstly, metastatic cancer cells can seed new foci and grow into new tumors in target organs, even after excision of the primary tumor due to early dissemination and seeded metastasis [10]. Secondly, metastatic cancer cells exhibit enhanced survival ability and resistance to most chemo- and radiotherapy that are highly efficient in certain primary nonmetastatic cancer cells [11, 12].

Ubiquitous calcium ions (Ca^2+^) are major second messengers and are critical to diverse physiological cellular events. Remodeling Ca^2+^ signals have been demonstrated to be essential in numerous diseases, including cancer [13–15]. In addition to chemoresistance, Ca^2+^ is also of importance in cancer metastasis, as proven decades ago [16, 17]. For example, Ca^2+^ is essential for focal adhesion turnover by regulating tyrosine kinase focal adhesion kinase (FAK) [13]. Focal adhesion turnover is critical in cycles of adhesion and detachment. It forms the traction points through which a cell moves forward and retracts the rear [18]. The intracellular distribution of Ca^2+^ is delicately balanced by several channel/pump/transporter families, including dozens of proteins located on the plasma membrane, endoplasmic reticulum (ER), mitochondria, and lysosomes. The signals of regional Ca^2+^ oscillations are decoded by numerous Ca^2+^-binding proteins with different distributions and affinities for Ca^2+^, further inducing a signaling cascade to regulate cell activity [17]. With the incessant discoveries of metastatic mechanisms, Ca^2+^-binding proteins, and Ca^2+^ channels/pumps/transporters, the roles of Ca^2+^ signaling in cancer metastasis have been increasingly revealed, serving as promising biomarkers for the prognosis and therapeutic targets of cancer metastasis.

## Basic process of tumor metastasis

Solid tumor metastasis is a complicated multistep and inefficient process. It includes separation and migration from primary tumors, invasion of local tissues, intravasation into blood or lymphatic vessels for transmission, extravasation into distant tissues and growth into macroscopic tumors [9]. Both cell-autonomous and noncell-autonomous mechanisms are crucial for successful cancer metastasis [19]. The plasticity of cancer cells, including their morphology and metabolism, is one major reason for successful cancer metastasis. For example, cells usually require the formation of lamellipodia at their leading edge, cycles of focal adhesion turnover, cell body contraction, and tail retraction [20–22]. Furthermore, intervention stresses, including radio- [23] and chemo-therapy [11], can also cause cancer metastasis.

The “amoeboid” form of invasion means that individual cancer cells with morphological plasticity can pass through existing interstices in the extracellular matrix (ECM). This movement differs from that of collective and mesenchymal invasion, both of which are dependent on the ability of the cancer cells to clear a path [24, 25]. Collective metastasis involves clusters of cancer cells invading adjacent tissues and spreading to distal organs [26]. Some studies demonstrate that multicellular tumor cell clusters, not single cell, present tumor invasive fronts, systemic circulation, and colonization of distant organs. Within these clusters, cancer cells may communicate and exchange signaling factors to promote metastasis [6, 27]. Mesenchymal invasion through the EMT program is the best characterized form of metastasis. In this process, cancer cells remodel the pattern of cytoskeletal proteins and adhesion molecules, and they further acquire stem features. E-cadherin, a classical and critical adhesion molecule, is lost in EMT. This loss favors cancer cells escape from endothelial cell (EC) sheets and promotes metastasis [20].

Tumor cells penetrating tissues through confining spaces must undergo extensive deformation, including its nucleus. However, this deformation can cause localized loss of nuclear envelope (NE) integrity and DNA damage, which may be a weakness of metastatic cancer cells [28]. Cancer cells exhibit increased chromatin accessibility during metastatic progression, which enhances prometastatic gene expression and promotes the metastatic ability of cancer cells [29]. Synonymous mutations may specifically regulate the expression of proteins that favor metastasis. Upregulation of tRNA^Glu^UUC and tRNA^Arg^CCG promotes breast cancer metastasis, with tRNA^Glu^UUC promoting metastatic progression by directly enhancing exosome component exosome component 2 (EXOSC2) and glutamate receptor interacting protein 1 (GRIP1) associated protein 1 (GRIPAP1) protein expression [30]. The expression of genes coding ribosomal proteins (RPs) and regulators of translation is also increased in breast circulating tumor cells (CTCs). RPL15 (eL15), a component of the large ribosomal subunit, increases the translation rate of other RPs and cell cycle regulators, leading to breast cancer cells metastatic growth in multiple organs [31].

## Mechanisms of cancer cells metastasis

### Separation and migration from primary tumors

Dissemination and metastasis may occur at an early tumor stage or even precede primary tumor formation [10, 32, 33]. One force that drives cells disseminating from tumors is the repulsive effect, which is also called contact inhibition of locomotion (CIL). CIL suppresses forward locomotion during cell-cell contact and redirects cell motility, which can enhance cancer invasiveness by favoring redirection of cancer cells into the stromal environment [34].

In primary tumor sites, cancer cells recruit myeloid-derived suppressor cells (MDSCs) to induce the acquisition of the EMT/stemness phenotype in cancer cells at the invasive cancer edge [35]. Ephrin type-A receptor 2 (EphA2) promotes membrane-anchored membrane type-1 matrix metalloproteinase (MT1-MMP) expression in invasive breast cancer cells. MT1-MMP in turn cleaves EphA2 and activates the EphA2/Src/RhoA axis, which induces actomyosin contractility and cell junction disassembly. These changes ultimately induce cell transition to rounded morphology, cell-cell repulsion, dissemination, and single-cell invasion [36].

### Crosstalk between cancer cells and circulatory systems

For seeding and growth at the secondary sites, cancer cells usually infiltrate into circulatory systems such as lymphatic and/or vascular vessels. CTCs are essential for distant metastasis of cancer, but only a small subset of CTCs can generate metastatic tumors [37–39]. This is because circulatory systems and distant organs are usually not hospitable to cancer cells, due to oxidative stress, anoikis, and the immunosurveillance system [40–42]. Melanoma cells with high metastatic ability preferentially upregulate monocarboxylate transporter 1 (MCT1) to consume more lactate, and cells with high MCT1 expression are more prominent in blood than in primary tumors. Selective inhibition of MCT1 does not affect the migration or invasion of melanoma cells or primary tumor growth, but it significantly reduces the frequency of circulating melanoma cells in the blood and cancer metastasis. This outcome is the result of MCT1 promoting cell survival during metastasis, partly by decreasing oxidative stress [37, 43]. Internalized β1-integrin localizes to autophagy-related endomembranes and triggers c-Met-sustained prosurvival in an adhesion-independent manner, preventing detached cancer cells from undergoing anoikis. This mechanism gives cancer cells sufficient time to land successfully [44].

#### Crosstalk between cancer cells and the lymphatic system

Lymphatic and vascular systems are the major access for cancer cells destined for distal metastasis by increased permeability [45], lymphoid hyperplasia [46], or angiogenesis [47]. Interestingly, cancer cells preferentially metastasize regionally through the lymphatic system before metastasizing to distal organs via blood. This outcome is partially the result of lymph fluid containing a high level of oleic acid, which protects cancer cells from ferroptosis in an acyl-coenzyme A synthetase long-chain family member 3 (ACSL3)-dependent manner [42]. Vascular endothelial growth factor-D (VEGF-D) produced by cancer cells inhibits prostaglandin degradation and results in the dilation of collecting lymphatic vessels, which ultimately promotes cancer metastasis [48]. Melanoma cells secrete midkine to induce distal premetastatic niches by driving lymphangiogenesis in a paracrine-dependent manner [46]. Cancer cells also produce interleukin-6 (IL-6) to activate signal transducer and activator of transcription 3 (STAT3) to induce hypoxia-inducible factor-1alpha (HIF-1α), VEGF, and C-C motif ligand (CCL) 5 expression in lymphatic ECs (LECs) within premetastatic niches to promote metastasis [49]. IL-10 derived from tumor-associated macrophages (TAMs) increases the expression of specificity protein 1 (Sp1) in LECs in a hypoxic TME. This Sp1 expression in turn induces the production of CCL1 in activated LECs to recruit TAMs and cancer cells, resulting in lymphatic vessels encapsulated by TAMs (LVEM) to promote cancer metastasis [50].

#### Crosstalk between cancer cells and the vascular system

Patrolling monocytes in blood possess early interactions with metastatic cancer cells. They can scavenge cancer cells from the vasculature and promote natural killer (NK) cell recruitment and activation [41]. Platelet-secreted transforming growth factor β (TGFβ) is necessary but not sufficient for the extravasation and subsequent metastatic niche formation of CTCs. Furthermore, platelets directly interacting with CTCs activate nuclear factor-kappa B (NF-κB) signals and induce the EMT in cancer cells, which in cooperation with TGFβ signaling facilitates metastasis [51, 52].

Cancer cells can recruit ECs and initiate angiogenesis [47]. Chemotherapy can also enhance angiogenesis to promote metastasis [53]. In addition to recruiting myeloid cells, tumor cell-derived CCL2 activates C-C motif receptor (CCR) 2 on ECs, inducing vascular permeability and subsequent cancer cell extravasation [45]. In brain cancer metastasis, melanoma cells increase blood-brain barrier (BBB) permeability [54]. Amyloid precursor proteins from cancer cells induce endothelial necroptosis via death receptor 6 (DR6) on ECs to enhance extravasation [55]. The intracellular domain of Notch1 receptors (N1ICDs) are activated in ECs in both primary tumors and premetastatic niches. N1ICDs ultimately induce EC senescence, neutrophil infiltration, tumor cells adhesion to the endothelium, intravasation, and metastasis [56].

Some cancer cells preferentially attach to the vascular network and migrate through endothelial layers by adopting a spindle-shaped morphology. They invaginate into the endothelial network by forming nanoscale membrane bridges between metastatic cancer cells and ECs to transfer signaling factors. This signaling leads to a cancer cell-induced pathological endothelium and metastatic foci *in vivo* [57].

Metastatic cancer cells that intravasate into the vasculature secrete Serpin Family E Member 2 (SERPINE2) and secretory leukocyte protease inhibitor (SLPI) to promote metastasis. These factors are required to program cancer cells for vascular mimicry, a program in which cells differentiate into endothelial-like cells and form extracellular-matrix-rich tubular structures to carry blood from the vasculature to hypoxic regions of the tumor [58]. A novel theory explaining cancer spread is called angiotropic migration, which is derived from a program initially involved in embryogenesis. In cancer metastasis, cancer cells attach to the abluminal surfaces of blood vessels and spread via continuous migration, competing with pericytes and replacing them, i.e. pericyte mimicry. This manner of spreading has been demonstrated in several types of cancer, including melanoma, glioma, colon, and breast cancer [59].

### Metastatic colonization

Metastatic cancer cells at secondary sites may die, be cleared, grow into macrometastases, or remain dormant only to grow into micrometastases decades after seeding [9, 32]. The reasons for the dormancy of cancer cells may be attributed to unfit proliferation conditions, such as nutrient starvation or inhibition by the immune system, or they may acquire suppressive factors from the primary tumor, or lack the ability to activate angiogenesis [60]. For example, breast cancer cells can directly engulf mesenchymal stem cells to enhance their prosurvival ability, but they then turn dormant [61].

Bone marrow-derived myeloid cells can become central nervous system (CNS) myeloid-like cells once they translocate to the brain. CNS-native myeloid cells display downregulation of CX3CR1, which enhances C-X-C motif (CXC) chemokine ligand (CXCL)10 signaling to foster an immunosuppressive niche and promote brain metastasis of cancer cells [62]. In addition, astrogliosis and neuroinflammation are instigated in the early stage of metastasis to promote the initial growth of metastatic melanoma cells in the brain [54].

#### Hospitable destinations: the liver and lung

Thrombopoietin-mediated activation of lysine degradation reduces oxidative stress via the Lys-Glu pathway and induces c-myc to recruit chromatin modifiers to regulate gene expression in cancer cells. This process enhances the production of acetyl-CoA and is essential for thrombopoietin-binding receptor-positive colorectal cancer cells spread in liver metastasis. The liver is the major organ producing thrombopoietin, which is a niche component that is critical for cancer cell colonization. Thrombopoietin production is probably why the liver is a preferred target organ of metastatic cancer cells [63].

The lung with high oxygen is also a favored target of metastatic cancer cells. Prolyl hydroxylase (PHD), an oxygen sensor, is intrinsically and functionally redundant in lung T lymphocytes. PHD inhibits HIF accumulation to suppress glycolytic metabolism. This suppression leads to a pulmonary T helper (Th)-1 cell response, promotes CD4^+^-regulatory T cell (Treg) induction, and restrains CD8^+^ T cell effector function, ultimately resulting in an immunosuppressive microenvironment that gives license to tumor colonization at the lung [64].

#### Adaptation to the target organ microenvironment

Efficiently taking advantage of the homing organ microenvironment is especially important for successful metastasis of cancer cells. In addition to sensing the level of Rac activity in adjacent cells to organize individual cells in cell clusters, Rab11b is necessary for metastatic breast cancer cells to adapt to the brain microenvironment, since Rab11b manipulates the expression of critical proteins at the cancer cell surface. For example, Rab11b regulates the recycling of integrin β1, which allows cancer cells to engage with the brain ECM efficiently to activate mechanotransduction signaling [65, 66]. To adapt to hepatic hypoxia, metastatic colon cancer cells (CRCs) import extracellular phosphocreatine, which is mediated by the creatine transporter SLC6A8, to generate intracellular ATP [67]. Ovarian cancer cells that metastasize to the omentum utilize fatty acids produced by adipocytes to meet their energic requirement via upregulating fatty acid-binding protein (FABP) 4 [4].

Brain astrocytes secrete exosomes containing miRNAs that inhibit phosphatase and tensin homolog (PTEN) expression in metastatic cancer cells. This process is reversible since cancer cells restore PTEN expression after they leave the brain. Loss of PTEN in cancer cells leads to the upregulated secretion of the chemokine CCL2, which recruits ionized calcium binding adaptor molecule 1 (IBA1)-expressing myeloid cells. This process ultimately promotes the outgrowth of brain metastatic tumor cells via enhanced proliferation and attenuated apoptosis [68].

#### Preparation and inducing transformation of the soil

Most cancer cells are poorly adapted to the microenvironment of the premetastatic tissue where they land, at least initially. The transcriptional and proteomic diversity between micrometastatic and primary tumor cells is universal. Cancer cells that have undergone the EMT may undergo the mesenchymal-epithelial transition (MET) to restore efficient proliferation ability, which can be induced by granulocytic MDSCs (gMDSCs) that are recruited by cancer cells at metastatic sites [35, 69, 70]. Cancer cells can inhibit Th-1 polarization or decrease CD4^+^ T cell levels in the metastatic TME to favor cancer cells survival [7, 71].

Cancer cells may remodel the ECM to form a premetastatic niche by secreting extracellular vesicles (EVs) in advance to escape dormancy at metastatic sites [72–74]. Tumor cells can prepare the premetastatic niche by secreting exosomes, which can be taken up by specific resident cells at their predicted destination. For example, the secretome of hypoxic primary cancer contains lysyl oxidase, which facilitates osteolytic lesion formation to promote CTCs colonization and metastasis formation in bone [75]. Exosomes from lung-, liver- and brain-tropic tumor cells fuse preferentially with lung fibroblasts and epithelial cells, liver Kupffer cells, and brain ECs, respectively. These exosomes determine the target organ of cancer cells; for example, exosomes from lung-tropic model cells redirected the metastasis of bone-tropic tumor cells because they transport specific integrins to the distal organ, where they activate Src and upregulate the expression of promigratory and proinflammatory S100 molecules. In addition, α6β4 and α6β1 are associated with lung metastasis, while αvβ5 is linked to liver metastasis [76].

Once CTCs arrive at the target organ, they lodge in capillaries and shed microscale blebs with diameters of ~ 5 μm into the vasculature. Most of these microparticles preferentially attach to the vasculature or independently migrate along the inner walls of vessels instead of dispersing in the blood flow. These microparticles are ingested by they recruit waves of distinct myeloid cell subsets. The cells in the early waves facilitate the survival of CTCs, while a small population of resident conventional dendritic cells in the last waves interact with CTCs and exert antimetastatic effects [77].

Phosphorylation of adenosine monophosphate (AMP)-activated protein kinase (AMPK)α is upregulated in metastatic tumors. Deletion of AMPKα impairs lung metastasis without affecting primary tumor growth. This effect is due to the ability of AMPK to phosphorylate the catalytic α subunit of PDHc (PDHA) to drive the tricarboxylic acid (TCA) cycle, which renders cancer cells resistant to metabolic and oxidative stress [78]. Fructose can promote metastasis of colon and breast cancers [79, 80]. CRCs that metastasize to the liver display upregulation of aldolase B (ALDOB). ALDOB promotes fructose metabolism, which is essential for the growth of CRCs in the liver. More importantly, CRC cells that are directly injected into the liver also display an increase in ALDOB. This finding directly demonstrates that reprograming metabolism occurs both at the premetastatic stage and when cancer cells land on new organs [79].

### Crosstalk between cancer cells and the TME

Communications between cancer cells and the TME are pivotal in cancer metastasis. The TME, including the ECM, bacteria, fibroblasts, mesenchymal stem cells, and macrophages, is essential for the phenotype of tumor [12, 81, 82]. Several types of cells derived from bone marrow, including macrophages, neutrophils, mast cells, and myeloid progenitors, are involved in pathological angiogenesis. In addition to reducing the effect of drugs targeting vasculature EC signals, they can facilitate local invasion [9, 83]. Cancer cells recruit gMDSCs and monocytic MDSCs (mMDSCs) for infiltration. The mMDSCs promote cancer cells dissemination by inducing EMT/stemness, while gMDSCs promote metastatic growth by reverting EMT/stemness [35]. Bacteria were firstly detected in human tumors more than 100 years ago. Intracellular bacteria are present in both cancer and immune cells [84]. Exosomes from those bacteria deliver factors such as IL-8 to noninfected cancer cells to increase the metastasis rate [1].

#### Cancer-associated fibroblasts

Cancer-associated fibroblasts (CAFs) can produce exosomes that transfer bioactive factors such as miRNA to induce the EMT and stemness of cancer cells [12]. Cancer cells undergoing the EMT preferentially localize to the leading edge of primary tumors, resulting in their positioning adjacent to the stroma, which includes CAFs [85]. EphA-mediated CIL promotes the formation of disseminated tumor cells (DTCs) [36, 86]. Furthermore, EphB2-induced EphB3/EphB4/Cdc42 signaling tightens the interaction between DTCs and CAFs, allowing for invasiveness and metastasis [86]. CAFs generate tracks to facilitate collective invasion by remodeling protease- and force-mediated matrix [2]. CAFs produce ligands that correspond to receptors expressed by cancer cells to promote metastasis [85]. For example, TGFβ derived from cancer cells enhances the secretion of IL-11 in CAFs. IL-11 in turn activates GP130/STAT3 in cancer cells to initiate metastasis [85]. CAFs can act as damage-associated molecular pattern (DAMP) sensors. Activation of nucleotide-binding domain and leucine-rich repeat containing (NLR) family pyrin domain containing 3 (NLRP3) signaling in CAFs increases the production of IL-1β. IL-1β facilitates breast cancer cells metastasis in the lung by modulating the immune cell milieu at the metastatic site and the expression of adhesion molecules [87].

#### TAMs

TAMs secrete TGFβ1, EGF and matrix-degrading enzymes, including Ca^2+^-dependent MMPs [88, 89]. Metastatic double-negative prostate cancer (DNPC) cells express high level of CCL2 to recruit M2-like TAMs and Tregs, which enhances metastasis initiation in conjunction with immunosuppression and neoangiogenesis [39]. In addition to promoting angiogenesis, angiotensin (Ang) II-activated ECs also produce CCL2. This production recruits additional CCR2^+^ macrophages to infiltrate tumors, which ultimately promotes metastasis [90]. TAMs are negatively correlated with the survival of hepatocellular carcinoma (HCC) patients, since these TAMs secrete TGFβ1 to induce the EMT and confer higher invasiveness in cancer cells [91]. Interestingly, cancer cells can produce a series of factors, including IL-4, colony stimulating factor-1 (CSF-1) and platelet-derived growth factor-BB (PDGF-BB), directly or indirectly in a pericyte- and fibroblast-derived IL-33-dependent manner to recruit TAMs, and thus promote metastasis [9, 92].

#### Mesenchymal stem cells

Mesenchymal stem cells (MSCs) are usually involved in maintaining tissue architecture and mediating pathological stromal responses during injury repair. Interestingly, cancer cells produce soluble factors that recruit MSCs from the bone marrow to the primary tumor site. Intratumor MSCs secrete CCL5 to stimulate the invasive behavior of cancer cells [93, 94]. Moreover, CXCL16 produced by cancer cells binds to CXC receptor (CXCR) 6 on MSCs, leading to the transition of MSCs to CAFs and the production of CXCL12. CXCL12 further binds to CXCR4 on cancer cells, inducing EMT and promoting metastasis [95]. In addition to TAMs, a small population of breast cancer cells can engulf MSCs to promote self-renewal, mesenchyme-like and metastatic properties [94, 96].

### Communications between cancer cells

Communications between cancer cells depend on direct interactions or EVs, including exosomes, to transfer biomolecules such as proteins and nucleic acids [97]. Cancer cells undergoing EMT can produce the EMT-inducing transcription factor Six1 to promote the metastasis of non-EMT cancer cells [98]. Collective metastasis is also dependent on the EMT. Clusters of cancer cells internalize N-cadherin, which induces the acquisition of only a partial mesenchymal phenotype characterized by an increase in tissue fluidity that similar to a solid-like-to-fluid-like transitional state. This transitional state allows cells to migrate under physical constraints without abolishing the cell cooperation required for collectiveness [99]. This enhanced cell metastatic potential is associated with poorer prognosis in cancer patients [6, 27]. These tumor cell clusters preferentially display high level of the epithelial cytoskeletal protein keratin 14 (K14), desmosome and hemidesmosome adhesion complex genes but are depleted of major histocompatibility complex (MHC) class II genes [100].

Cancer cells within multicellular clusters can form nano alumina to construct their own internal microenvironment, from which they exchange and concentrate growth factors such as epigen to drive metastatic outgrowth [6]. Although E-cadherin has been widely demonstrated to inhibit cancer metastasis, E-cadherin is intriguingly required for the metastasis of invasive ductal carcinoma (IDC). This requirement is based on that E-cadherin enable cancer cells to form collective metastasis by reducing reactive oxygen species (ROS) and preventing cancer cells from apoptosis, which is especially critical during systemic dissemination and early seeding [39]. Clusters, usually containing as many as 20 cells, that are too large to pass through narrow vessels can reversibly reorganize into single-file chain-like geometries that reduce their hydrodynamic resistances, allowing them to successfully traverse 5~10 μm constrictions, even in whole blood [101]. Correspondingly, PEP06 polypeptide 30, a cluster-dissociating agent, inhibits cancer metastasis by manipulating the EMT, the α_v_ integrin/FAK/Src axis, and E-cadherin-based intercellular junctions [102, 103].

### Reprogramming metabolism

Antioxidants *N*-acetylcysteine and vitamin E reduce ROS, free heme level, and stabilize the transcription factor BACH1, resulting in glycolysis-dependent metastasis of lung cancer cells [104]. This finding suggests that oxidative stress may have an inhibitory effect on metastasis at the initial stage. Metastatic melanoma cells but not primary melanoma cells are capable of engulfing and digesting live autologous melanoma-specific CD8^+^ T cells to increase their survival, particularly under nutrient-stress conditions [105].

Some cancer stem cells (CSCs) do not display high levels of mesenchymal genes but express the high level of the fatty acid receptor CD36. These CSCs are critical for metastasis initiation and rely particularly on dietary lipids to promote metastasis [106]. Although the primary and lymph node metastases of melanoma cells display a similar level of cholesterol, metastatic cells in lymph nodes can probably produce or recruit more bile acids to activate vitamin D receptor/YAP, which promote metastatic cells adaptation to their new microenvironment [107].

Aerobic glycolysis (the Warburg effect) has been widely used as a marker of cancer [9, 108]. FABP7 is increased in patients with breast cancer metastases in the brain. It inhibits fatty acid oxidative phosphorylation (OXPHOS) and enhances the formation of lipid droplets, which facilitates HER2^+^ breast cancer metastasis to the brain [109]. Moreover, DTCs may reprogram their metabolism to survive and colonize at metastatic sites. Micrometastatic triple-negative breast cancer (TNBC) cells in the lungs and lymph nodes upregulate mitochondrial OXPHOS, the pharmacological inhibition of which profoundly reduces lung metastasis [38]. CTCs display high expression of peroxisome proliferator-activated receptor-gamma 1α (PGC-1α), which is regulated by Ca^2+^ and is necessary for intravasation of cancer cells into the circulatory systems. PGC-1α enhances mitochondrial biogenesis and respiration coupled with the remodeling of actin cytoskeleton signals. Silencing of PGC-1α ultimately decreases the aggressive properties of cancer cells, such as metastatic lung colonization and nodule formation [38, 110].

### Escape from immunosurveillance

Leukocyte immunoglobulin-like receptor B4 (LILRB4) is exclusively expressed on monocytic cells. It inhibits T cell proliferation and T cell-mediated antitumor immunity meanwhile promoting acute myeloid leukemia (AML) cell migration and tumor infiltration [111]. Metastatic castration-resistant prostate cancer (mCRPC) cells induce an increase in the population of osteoclasts in bone marrow. Osteoclasts produce TGFβ, which inhibits Th-1 polarization in the metastatic TME and is the cause of the poor response of metastatic cancer to immune checkpoint therapy [71]. Cancer cells that have metastasized to the liver can recruit macrophages and siphon-activated antigen-specific T cells. These macrophages display high expression of FasL, which diminishes CD4^+^ T cells through their direct contact, which induces FasL/Fas-dependent apoptosis, resulting in systemic immunosuppression [7]. PHD restrains CD8^+^ T cell effector function, also leading to an immunosuppressive microenvironment that favors cancer cell colonization at the lung [64].

## Roles of calcium signaling in cancer metastasis

Ca^2+^, a ubiquitous second messenger, is critical to cancer development, including metastasis and therapy resistance [17]. Ca^2+^ has been widely demonstrated to manipulate events critical to metastasis, including the EMT [112], invadosome formation, ECM degradation [113] and angiogenesis [114]. Serum Ca^2+^ level is positively associated with the risk of node metastasis in endometrial and breast cancer [115, 116], but low serum calcium level indicates poor distant metastasis-free survival of patients with nasopharyngeal carcinoma [117].

Cancer cells contacting with ECs induces oscillatory intracellular and intercellular Ca^2+^ waves in ECs that generated from the contact site. It coupled with ECs retraction that may be responsible for cancer metastasis [118]. Direct current electric field (dcEF)-mediated epidermal growth factor receptor (EGFR) polarization and Ca^2+^ influx promote cancer cell electrotaxis [119]. β_2_-adrenoceptor initiates a cAMP-Ca^2+^ feedforward loop that enhances the invasion of breast cancer cells [120]. Metformin inhibits fibrosarcoma invasion and migration by reducing MMP9 activity in a Ca^2+^-dependent manner [121]. Several Ca^2+^ channel blockers have been demonstrated to inhibit cancer cell metastasis in diverse ways. Nifedipine and diltiazem reduce cancer cells adhesion to ECs and the metastasis rate by remodeling the tumor cell cytoskeleton, affecting cell mobility and inhibiting tumor cell-induced platelet aggregation [122].

### Ca^2+^-dependent proteins

Numerous Ca^2+^-dependent proteins containing Ca^2+^-binding sites, such as calmodulin (CaM) [123, 124], Ca^2+^/CaM-dependent kinase (CaMK) [125], calreticulin [126], Ca^2+^ sensor [127], Ca^2+^-sensing receptor (CaSR) [128], calpain [129], calcineurin [130], myosin light-chain kinase (MLCK) [131] and phospholipase S100 family proteins [132], have been widely proven to be vital to cancer metastasis. CaM, with four EF-hands, is one of the most important Ca^2+^ signal-decoding proteins. It can rapidly redistribute to subcellular compartments in response to various signals. Migration signals trigger a spatiotemporal redistribution of CaM to the leading edge of the migrating cell; for example, EGF induced CaM redistribution from the nucleus to the cytoplasm to activate invadopodia-associated proteins [123, 133]. Calreticulin can inhibit ER stress to inhibit EMT, which promotes cancer cells liver metastasis [126]. CaSR located on the plasma membrane senses extracellular Ca^2+^. CaSR induces activation of AKT/β-catenin, leading to cancer metastasis [128, 134]. Calpain cleaves talin, resulting in more rapid adhesion disassembly rates [129]. Neuronal Ca^2+^ sensor 1 (NCS1) promotes the motility of cancer cells by localizing to the cell leading edge [135].

S100A4 reduces the expression of E-cadherin and β-catenin in cancer cells, while S100A4^+^ stromal cells enhance the stem cell-like phenotype of tumor cells [136, 137]. S100A9 can be secreted by TAMs, which is increased in HCC-related TAMs and HCC cells. It enhances the stem cell-like and metastatic properties of HCC cells by activating NF-κB and recruiting more macrophages to infiltrate tumors [138, 139]. Ca^2+^-activated potassium channels promote the proliferation and migration of endometrial and breast cancer cells, while they also mediate blood-brain tumor barrier opening to promote brain metastasis [140–142].

Limited by the scope of this brief review to cover all Ca^2+^-dependent proteins and protein-mediated Ca^2+^ currents, we mainly focus on the proteins critical for Ca^2+^ currents that have also been reported to be involved in cancer metastasis (Table 1).

**Table 1.** Representative studies about Ca^2+^ channels/pumps/uniporters in cancer metastasis

**Channels/Pumps/Uniporters**		**Alteration**	**Events/Mechanisms**	**Cancer type**	**Reference**
VGCC	Cav1.2	↑	-	CRC	[143]
	Cav1.3	↑	-	Breast cancer, endometrial carcinoma	[144, 145]
	Cav2.2	↑	EMT	Breast cancer	[146]
	Cav3.1/3.2	-	-	Glioblastoma, RAF^V600E^ melanoma	[147, 148]
CRAC	-	-	Upregulation of MFAP5 derived from CAFs increases SOCE in cancer cells	Ovarian cancer	[149]
	Orai1	↑	Focal adhesion turnover, invadopodium formation, EMT and extracellular matrix degradation	Glioma, melanoma and breast cancer	[13, 150–153]
	Orai2	↑	Focal adhesion disassembly	GC	[154]
	Orai3	-	Neuroendocrine-to-mesenchymal transition	Gastro-enteropancreatic neuroendocrine tumor	[151]
	STIM1	↑	Focal adhesion turnover and invadopodium formation	Melanoma, gastric and breast cancer	[13, 153, 155]
	STIM2	↑	EMT	Breast cancer	[8]
	MS4A12	↑	-	Colon cancer	[156]
TRP channel	TRPC1	↑	EMT	Breast cancer	[157]
	TRPC3	↑	-	Melanoma	[158]
	TRPC4	-	-	Medulloblastoma	[159]
	TRPC5	↑	EMT	Colon cancer	[160]
	TRPC6	↑	EMT	Breast and gastric cancer	[161–163]
	TRPM2	-	EMT	Gastric cancer	[164]
	TRPM3	↑	EMT	Renal cell carcinoma	[165]
	TRMP4	↑	EMT	Prostate cancer	[166, 167]
	TRPM5	-	MMP9	Melanoma	[168]
	TRPM7	↑	EMT	Nasopharyngeal carcinoma, ovarian and breast cancer	[169–171]
	TRPM8	↑	EMT	Colon cancer	[172]
	TRPML1	-	Lysosomal exocytosis	Hepatocellular carcinoma	[173]
	TRPP2	↑	EMT	Laryngeal squamous cell carcinoma	[174]
	TRPV1	↓	Peritoneal dissemination	GC	[175]
	TRPV2	↑	-	Prostate and esophageal cancer	[176]
	TRPV4	↑	Matrix stiffness and EMT	Endometrial and breast cancer	[177, 178]
		↓	Tumor vessel integrity	Prostate cancer	[179]
	TRPV5	↓	-	Renal cell carcinoma	[180]
	TRPV6	↑	-	Prostate cancer	[181]
ATP dependent pumps	PMCA4	↓	EMT	GC	[182]
	SERCA	-	Focal adhesions	Glioblastoma	[183]
	SERCA2	↑	-	Colorectal cancer	[184]
	SERCA3	↓	-	Colorectal adenoma-adenocarcinoma	[185]
	P2X7	↑	EMT and formation of filopodia	Melanoma and colon cancer	[186–188]
	P2Y	-	-	Colon cancer	[187]
	P2Y2	-	EMT	Prostate cancer	[189, 190]
	P2Y6	-	Filopodia and focal adhesions	Lung cancer	[191]
	P2Y12	-	Positive regulation of Akt in platelets	-	[192]
MCU complex	EMRE	↓	-	TNBC	[193]
	MCU	↑	ROS, MVD, tube formation and sprouting capacity	Hepatocellular carcinoma and TNBC	[194–196]
	MCUb	↓	ROS	TNBC	[196]
	MICU1	↑	ROS	TNBC	[193]
		↓	ROS	Hepatocellular carcinoma	[194]
	MICU2	↑	ROS	TNBC	[193]
	MCUR1	↑	EMT	HCC	[197]
TPCs	Tpc2	↓	EMT	Melanoma	[198]
IP_3_R	IP_3_R-3	↑	-	Colorectal cancer	[199]
RyR	RyR2	↓	-	Thyroid carcinoma	[200]

↑: indicates increased; ↓: indicates decreased; -: indicates not availab; VGCC: voltage-gated Ca^2+^ channel; MFAP5: microfibrillar-associated protein 5; SOCE: store-operated Ca^2+^ entry; STIM: stromal interaction molecule; TRP: transient receptor potential; TRPC: TRP canonical; TRPM: TRP melastatin; TRPML1: Mucolipin TRP channel 1; TRPP2: TRP polycystin 2; TRPV: TRP vanilloid; PMCA: plasma membrane Ca^2+^ ATPase; GC: gastric cancer; SERCA: (Sarco)-ER Ca^2+^ ATPase; MCU: mitochondrial calcium uniporter; EMRE: essential MCU regulator; MCUb: MCU dominant negative beta subunit; MICU: mitochondrial calcium uptake; MCUR1: MCU regulator 1; TPC: two-pore channel; IP_3_R: inositol 1,4,5-trisphosphate receptor; RyR: ryanodine receptor; MVD: microvessel density

### VGCCs

VGCCs comprise five subtypes and ten members. Endostatin, a commercial agent targeting angiogenesis, reduces glioblastoma cells migration by directly inhibiting T-type VGCCs [147]. Cav1.3 expression is increased in breast cancer, atypical hyperplasia, and endometrial carcinoma tissues. It partially mediates estrogen-induced Ca^2+^ influx and endometrial carcinoma cells migration [144, 145]. Ca^2+^ is increased at filopodia tips and correlated with filopodia stabilization. Filopodia stabilization leads to focal adhesion formation, which is critical to the migration of cells. L-type Ca^2+^ channel blockers, such as amlodipine, besylate, felodipine, manidipine, and cilnidipine, potently inhibit L-type Ca^2+^ channel/calpain-1-mediated filopodia formation, which efficiently reduces the cancer cells migration and invasion rates [145]. The Cav1.2 level is increased in CRCs. Nifedipine, another L-type Ca^2+^ channel blocker, inhibits CRC migration and PD-1 via Cav1.2/Ca^2+^-mediated activation of nuclear factor of activated T cell 2 (NFAT2). The combination of nifedipine with an anti-PD-1 antibody significantly reduces cancer cell liver metastasis and the PD-1^+^ CD8^+^ T cells level among tumor-infiltrating lymphocytes [143].

### Ca^2+^ release-activated Ca^2+^ channels

Orai proteins located on the cell membrane are the core components of Ca^2+^ release-activated Ca^2+^ channels (CRACs) that mediate SOCE. Upon ER Ca^2+^ depletion, STIM activates Orai to mediate Ca^2+^ influx. CRACs play critical roles not only in immune cells but also in cancer cells [13, 14, 201]. Chemoresistant ovarian cancer cells acquire enhanced migratory ability by increasing SOCE-mediated focal adhesion turnover [11]. ACE2 promotes metastasis of breast cancer by activating the PAK1/NF-κB/Snail1 axis via SOCE [202]. MFAP5 secreted by CAFs activates FAK/cAMP response element-binding protein (CREB)/troponin C type 1 (TNNC1) via SOCE to enhance cancer metastasis [149].

CRAC-mediated Ca^2+^ regulates invadopodium formation and ECM degradation [155, 203]. Orai1-medated SOCE promotes phosphorylation of proline-rich tyrosine kinase 2 (Pyk2), which regulates focal adhesion turnover and the EMT of glioma cells [150]. Elevation of Orai2 is positively correlated with lymph node metastasis of gastric cancer (GC). Orai2 induces FAK-mediated mitogen-activated protein kinases(MAPK)/extracellular signal-regulated kinase (ERK) activation and enhances focal adhesion disassembly at the rear edge of metastatic cancer cells [154]. The hEag1 K^+^ channels are essential for breast cancer cell migration because they promote Orai1-mediated Ca^2+^ current [204]. Inhibition of Orai or STIM decreases GTPase Ras- and Rac-mediated formation of new focal complexes at cell protrusions and disassembly of focal adhesions. This action ultimately inhibits breast cancer metastasis to the lung [13]. Orai3 and STIM2 also promote gastroenteropancreatic neuroendocrine and breast cancer metastasis, respectively [8, 151].

### TRP channels

The TRP channel superfamily comprises seven subtypes with approximately thirty members, most of which can mediate Ca^2+^ currents. TRP channels are activated by diverse stimuli, including intra- and extra-cellular messengers, temperature, pH, chemical, mechanical and osmotic stress, ROS, and intracellular Ca^2+^ stores [17]. The expression of phospholipid phosphatase 4 (PLPP4) protein is increased, and it promotes cancer cells metastasis probably by activating TRPC channels-mediated Ca^2+^ influx [205]. TRPC1 inhibits the PI3K/AKT pathway and EMT, leading to reduced migration and invasion rates of cancer cells [157]. TRPC5 is elevated in colon cancer, which promotes cancer cell EMT and metastasis via the HIF-1α/Twist axis. In addition to promoting the migration and invasiveness of renal cancer cells, the upregulated expression of TRPC6 is also required for translocation of Orai1 and Orai3 to the plasma membrane of metastatic breast cancer cells [161, 162]. *Helicobacter pylori*, a well-known major risk factor for GC, increases TRPC6 transcription and Ca^2+^ influx via the Wnt/β-catenin pathway to enhance GC cells migration and invasion [163].

Cancer cells with high level of TRPM2 channels are more susceptible to neutrophil cytotoxicity. A reduction in TRPM2 expression leads to decreased tumor growth but increased DTC seeding potential in the premetastatic lung [206]. Upregulation of TRPM7 expression is correlated with the EMT and metastasis of ovarian cancer via the Ca^2+^-related PI3K/AKT axis [171]. The level of TRPV1 channels is reduced in human GC tissues. This reduction leads to decreased Ca^2+^/calmodulin-dependent protein kinase kinase beta (CaMKKβ) activity and contributes to GC peritoneal dissemination [175]. TRPV4 or TRPV6 is increased in endometrial, breast or prostate cancer, which promotes cancer cells metastasis [177, 178, 181]. Deletion of TRPV4 increases p-VEGFR2^Y1175^ level and ECs migration [207]. However, deficiency of TRPV4 causes reduced vascular E-cadherin level and destabilizes tumor vessel integrity, leading to cancer cells lung metastasis [179].

### Adenine/uridine nucleotide-dependent proteins that mediate Ca^2+^ current

#### Plasma membrane Ca^2+^ ATPase

Reduction in PMCA4 level increases zinc finger E-box binding homeobox 1 (ZEB1) expression and the nuclear accumulation of NFAT isoform c1 (NFATc1). PMCA4 ultimately lead to advanced tumor-node-metastasis (TNM) stage and poor prognosis of GC patients. It also induces cancer cells transformation with elongated fibroblastoid morphology, reduced E-cadherin level and increased vimentin level, which can be inhibited by cyclosporine A [182].

#### SERCA

The miR-708 level is decreased in both lymph node and distal metastases, suggesting a cancer metastasis-suppressive role of miR-708. Neuronatin is a membrane protein in the ER with the ability to inhibit SERCA. Reduction in miR-708 level increases the expression of neuronatin, which further induces the elevation of intracellular Ca^2+^ level to promote cell migration and metastasis formation by activating the ERK/FAK axis [183]. Upregulation of SERCA2 in CRCs is positively related to serosal invasion and lymph node metastasis [184]. In contrast, the SERCA3 level is negatively related to lymphatic invasion of colorectal adenoma-adenocarcinoma [185].

#### Purinergic receptors

P2*7 (*indicates X or Y) receptors are highly expressed on LoVo and SW480 CRCs. Activation of P2*7 by ATP promotes CRCs metastasis by STAT3-dependent EMT [187]. P2X7 promotes cancer invasiveness by sustaining the activity of cell division cycle 42 (Cdc42) and promoting the acquisition of a mesenchymal phenotype [186]. P2Y mediates prostate cancer invasion via ERK1/2 and p38 or EMT/invasion-related genes [189, 190]. Activation of P2Y6 increases filopodia and the number of focal adhesions via Gαq/Ca^2+^/PKC and Gα_13_/Rho-associated protein kinase-dependent pathways to promote lung cancer cell migration [191]. Depletion of apoptosis signal-regulating kinase 1 (ASK1) reduces the phosphorylation of P2Y12 in platelets, which leads to defects in platelet aggregation and a reduction in tumor metastasis [192].

### Mitochondrial calcium uniporter

Mitochondria, major intracellular Ca^2+^ stores, are primary sources of ROS, which promote tumor metastasis. MCU located on the inner mitochondrial membrane, is a Ca^2+^-selective channel and critical for major Ca^2+^ influx into mitochondria [208]. MCU-mediated ATP production regulates profibrotic macrophage polarization [209]. cl-CD95L enhances the metastatic dissemination of TNBC cells by enhancing MCU-mediated Ca^2+^ current [210]. However, reduction in histidine triad nucleotide-binding 2 (HINT2) enhances pancreatic cancer lymph node metastasis, probably by increasing MICU1/2 and decreasing EMRE levels to inhibit MCU activity [193, 211].

MCU is significantly associated with cancer aggressiveness and is increased in diverse cancers, including TNBC, HCC, and GC [88, 194–196]. Elevation of MCU level enhances metastatic colonization and MVD in breast cancer. MCU also inhibits the selective sorting of miR-4488 to extracellular vesicles by manipulating Ca^2+^-dependent RNA-binding proteins (RBPs). This process leads to relief of C-X3-C motif chemokine ligand 1 (CX3CL1) repression to enhance tube formation capacity and sprouting capacity [195]. MCUb expression is decreased while that of MCU is increased in invasive TNBC and lymph node metastasis. Silencing MCU increases SOCE and the NADPH/NADH ratio while decreases ROS and HIF-1α levels [196]. MICU1 is decreased in metastatic HCC tissues, and MCU-mediated mitochondrial Ca^2+^ influx induces ROS/c-Jun N-terminal kinase (JNK) activation. This chain of events ultimately facilitates HCC intrahepatic and lung metastasis [194]. MCUR1 is significantly upregulated in metastatic HCC cells, which promote EMT and metastasis via the mitochondrial Ca^2+^-dependent ROS/Nrf2/Notch pathway [197].

### TPC

TPC family proteins are nicotinic acid adenine dinucleotide phosphate (NAADP)-gated Ca^2+^ release channels located on endosomes, lysosomes, and melanosomes. Reduction in TPC2 expression activates the YAP/TAZ axis to inhibit the expression levels of ORAI1 and PKC-βII, which is found in the metastatic tumors but not in the primary melanoma cells in patients [198].

### IP_3_R and RyR

The EGF-induced EMT is coupled with specific alterations in the mRNA level of ER-related Ca^2+^ channels/pumps, including SERCAs, IP_3_R, and RyR [212]. Trifluoperazine, an antipsychotic drug, can reduce glioblastoma invasion by binding with CaM to activate IP_3_R-mediated Ca^2+^ release from the ER [213]. Furthermore, IP_3_R-3 is increased in colon cancer, while RyR2 is decreased in thyroid carcinoma tissues, both of which are related to aggressiveness [199, 200].

## Conclusions

Metastasis is still a great challenge for improving cancer therapy, in which Ca^2+^-manipulated signals have been proven to be critical. Currently, although great progress has been made, the mechanisms underlying the metastatic cascade and functions of Ca^2+^ have not yet been fully elucidated. Most previous studies have focused mainly on Ca^2+^-dependent proteins or Ca^2+^ channels/pumps/uniporters in cancer cells, while only a few studies have investigated their functions in cancer stoma cells and the metastatic niche. There are still limited available drugs targeting Ca^2+^-mediated signals in the clinic or undergoing clinical trials for cancer metastasis therapy. Therefore, the development of drugs specifically targeting proteins that decoding calcium oscillation signals or mediating Ca^2+^ currents is highly desirable.

## Abbreviations

ALDOB: aldolase B
AMP: adenosine monophosphate
AMPK: AMP-activated protein kinase
Ca^2+^: calcium ions
CAFs: cancer-associated fibroblasts
CaM: calmodulin
CaSR: Ca^2+^ sensing receptor
CCL: C-C motif ligand
CIL: contact inhibition of locomotion
CRACs: Ca^2+^ release-activated Ca^2+^ channels
CRCs: colon cancer cells
CTCs: circulating tumor cells
CXC: C-X-C motif
CXCL: CXC chemokine ligand
DTCs: disseminated tumor cells
ECM: extracellular matrix
ECs: endothelial cells
EGF: epidermal growth factor
EMRE: essential MCU regulator
EMT: epithelial-to-mesenchymal transition
EphA2: Ephrin type-A receptor 2
ER: endoplasmic reticulum
ERK: extracellular signal-regulated kinase
FAK: focal adhesion kinase
GC: gastric cancer
gMDSCs: granulocytic MDSCs
HCC: hepatocellular carcinoma
HIF-1α: hypoxia-inducible factor-1alpha
IL-6: interleukin-6
IP_3_R: inositol 1,4,5-trisphosphate receptor
LECs: lymphatic ECs
MCT1: monocarboxylate transporter 1
MCU: mitochondrial calcium uniporter
MDSCs: myeloid derived suppressor cells
MET: mesenchymal-epithelial transition
MICU: mitochondrial calcium uptake
MMPs: matrix metalloproteinases
NF-κB: nuclear factor-kappa B
NK: natural killer
PGC-1α: peroxisome proliferator-activated receptor-gamma coactivator 1α
PHD: prolyl-hydroxylase
PMCA: plasma membrane Ca^2+^ ATPase
PTEN: phosphatase and tensin homolog
ROS: reactive oxygen species
RPs: ribosomal proteins
RyR: ryanodine receptor
SERCA: (sarco)-endoplasmic reticulum Ca^2+^ ATPase
SOCE: store-operated Ca^2+^ entry
STAT3: signal transducer and activator of transcription 3
TAMs: tumor-associated macrophages
TGFβ: transforming growth factor β
TME: tumor microenvironment
TNBC: triple negative breast cancer
TPCs: two-pore channels
TRP: transient receptor potential
TRPC: TRP canonica
TRPM: TRP melastatin
TRPV: TRPV vanilloid
VGCCs: voltage-gated Ca^2+^ channels

## Declarations

### Copyright

© The Author(s) 2021.

## References

[1] Guo S Chen J Chen F Zeng Q Liu WL Zhang G. Exosomes derived from Fusobacterium nucleatum-infected colorectal cancer cells facilitate tumour metastasis by selectively carrying miR-1246/92b-3p/27a-3p and CXCL16. Gut. Forthcoming 2021. 10.1136/gutjnl-2020-321187 33172926

[2] Gaggioli C Hooper S Hidalgo-Carcedo C Grosse R Marshall JF Harrington K Fibroblast-led collective invasion of carcinoma cells with differing roles for RhoGTPases in leading and following cells. Nat Cell Biol. 2007;9:1392–400. 10.1038/ncb1658 18037882

[3] Dai W Guo X Cao Y Mondo JA Campanale JP Montell BJ Tissue topography steers migrating Drosophila border cells. Science. 2020;370:987–90. 10.1126/science.aaz4741 33214282 PMC8103818

[4] Nieman KM Kenny HA Penicka CV Ladanyi A Buell-Gutbrod R Zillhardt MR Adipocytes promote ovarian cancer metastasis and provide energy for rapid tumor growth. Nat Med. 2011;17:1498–503. 10.1038/nm.2492 22037646 PMC4157349

[5] Zhang J Wang S Jiang B Huang L Ji Z Li X c-Src phosphorylation and activation of hexokinase promotes tumorigenesis and metastasis. Nat Commun. 2017;8:13732. 10.1038/ncomms13732 28054552 PMC5227066

[6] Wrenn ED Yamamoto A Moore BM Huang Y McBirney M Thomas AJ Regulation of collective metastasis by nanolumenal signaling. Cell. 2020;183:395–410.e19. 10.1016/j.cell.2020.08.045 33007268 PMC7772852

[7] Yu J Green MD Li S Sun Y Journey SN Choi JE Liver metastasis restrains immunotherapy efficacy via macrophage-mediated T cell elimination. Nat Med. 2021;27:152–64. 10.1038/s41591-020-1131-x 33398162 PMC8095049

[8] Miao Y Shen Q Zhang S Huang H Meng X Zheng X Calcium-sensing stromal interaction molecule 2 upregulates nuclear factor of activated T cells 1 and transforming growth factor-β signaling to promote breast cancer metastasis. Breast Cancer Res. 2019;21:99. 10.1186/s13058-019-1185-1 31464639 PMC6716836

[9] Hanahan D Weinberg RA. Hallmarks of cancer: the next generation. Cell. 2011;144:646–74. 10.1016/j.cell.2011.02.013 21376230

[10] Hosseini H Obradović MMS Hoffmann M Harper KL Sosa MS Werner-Klein M Early dissemination seeds metastasis in breast cancer. Nature. 2016;540:552–8. 10.1038/nature20785 27974799 PMC5390864

[11] Huang HK Lin YH Chang HA Lai YS Chen YC Huang SC Chemoresistant ovarian cancer enhances its migration abilities by increasing store-operated Ca^2+^ entry-mediated turnover of focal adhesions. J Biomed Sci. 2020;27:36. 10.1186/s12929-020-00630-5 32079527 PMC7033940

[12] Hu JL Wang W Lan XL Zeng ZC Liang YS Yan YR CAFs secreted exosomes promote metastasis and chemotherapy resistance by enhancing cell stemness and epithelial-mesenchymal transition in colorectal cancer. Mol Cancer. 2019;18:91. 10.1186/s12943-019-1019-x 31064356 PMC6503554

[13] Yang S Zhang JJ Huang XY. Orai1 and STIM1 are critical for breast tumor cell migration and metastasis. Cancer Cell. 2009;15:124–34. 10.1016/j.ccr.2008.12.019 19185847

[14] Feske S Gwack Y Prakriya M Srikanth S Puppel SH Tanasa B A mutation in Orai1 causes immune deficiency by abrogating CRAC channel function. Nature. 2006;441:179–85. 10.1038/nature04702 16582901

[15] Choi S Cui C Luo Y Kim SH Ko JK Huo X Selective inhibitory effects of zinc on cell proliferation in esophageal squamous cell carcinoma through Orai1. FASEB J. 2018;32:404–16. 10.1096/fj.201700227RRR 28928244 PMC6207365

[16] Alessandro R Masiero L Liotta LA Kohn EC. The role of calcium in the regulation of invasion and angiogenesis. In Vivo. 1996;10:153–60. 8744794

[17] Cui C Merritt R Fu L Pan Z. Targeting calcium signaling in cancer therapy. Acta Pharmaceutica Sinica B. 2017;7:3–17. 10.1016/j.apsb.2016.11.001 28119804 PMC5237760

[18] Webb DJ Parsons JT Horwitz AF. Adhesion assembly, disassembly and turnover in migrating cells--over and over and over again. Nat Cell Biol. 2002;4:E97–100. 10.1038/ncb0402-e97 11944043

[19] Fidler IJ Poste G. The “seed and soil” hypothesis revisited. Lancet Oncol. 2008;9:808. 10.1016/S1470-2045(08)70201-8 18672217

[20] Fife CM McCarroll JA Kavallaris M. Movers and shakers: cell cytoskeleton in cancer metastasis. Br J Pharmacol. 2014;171:5507–23. 10.1111/bph.12704 24665826 PMC4290699

[21] Li Y Zhang Z Zhou X Li L Liu Q Wang Z The oncoprotein HBXIP enhances migration of breast cancer cells through increasing filopodia formation involving MEKK2/ERK1/2/Capn4 signaling. Cancer Lett. 2014;355:288–96. 10.1016/j.canlet.2014.09.047 25304384

[22] Tang Y He Y Zhang P Wang J Fan C Yang L LncRNAs regulate the cytoskeleton and related Rho/ROCK signaling in cancer metastasis. Mol Cancer. 2018;17:77. 10.1186/s12943-018-0825-x 29618386 PMC5885413

[23] Harada H Inoue M Itasaka S Hirota K Morinibu A Shinomiya K Cancer cells that survive radiation therapy acquire HIF-1 activity and translocate towards tumour blood vessels. Nat Commun. 2012;3:783. 10.1038/ncomms1786 22510688 PMC3337987

[24] Sabeh F Shimizu-Hirota R Weiss SJ. Protease-dependent *versus*-independent cancer cell invasion programs: three-dimensional amoeboid movement revisited. J Cell Biol. 2009;185:11–9. 10.1083/jcb.200807195 19332889 PMC2700505

[25] Orgaz JL Pandya P Dalmeida R Karagiannis P Sanchez-Laorden B Viros A Diverse matrix metalloproteinase functions regulate cancer amoeboid migration. Nat Commun. 2014;5:4255. 10.1038/ncomms5255 24963846 PMC4118761

[26] Friedl P Wolf K. Tube travel: the role of proteases in individual and collective cancer cell invasion. Cancer Res. 2008;68:7247–9. 10.1158/0008-5472.CAN-08-0784 18794108

[27] Aceto N Bardia A Miyamoto DT Donaldson MC Wittner BS Spencer JA Circulating tumor cell clusters are oligoclonal precursors of breast cancer metastasis. Cell. 2014;158:1110–22. 10.1016/j.cell.2014.07.013 25171411 PMC4149753

[28] Denais CM Gilbert RM Isermann P McGregor AL te Lindert M Weigelin B Nuclear envelope rupture and repair during cancer cell migration. Science. 2016;352:353–8. 10.1126/science.aad7297 27013428 PMC4833568

[29] Denny SK Yang D Chuang CH Brady JJ Lim JS Grüner BM Nfib promotes metastasis through a widespread increase in chromatin accessibility. Cell. 2016;166:328–42. 10.1016/j.cell.2016.05.052 27374332 PMC5004630

[30] Goodarzi H Nguyen HCB Zhang S Dill BD Molina H Tavazoie SF. Modulated expression of specific tRNAs drives gene expression and cancer progression. Cell. 2016;165:1416–27. 10.1016/j.cell.2016.05.046 27259150 PMC4915377

[31] Ebright RY Lee S Wittner BS Niederhoffer KL Nicholson BT Bardia A Deregulation of ribosomal protein expression and translation promotes breast cancer metastasis. Science. 2020;367:1468–73. 10.1126/science.aay0939 32029688 PMC7307008

[32] Harper KL Sosa MS Entenberg D Hosseini H Cheung JF Nobre R Mechanism of early dissemination and metastasis in Her^2+^ mammary cancer. Nature. 2016;540:588–92. 10.1038/nature20609 27974798 PMC5471138

[33] Rhim AD Mirek ET Aiello NM Maitra A Bailey JM McAllister F EMT and dissemination precede pancreatic tumor formation. Cell. 2012;148:349–61. 10.1016/j.cell.2011.11.025 22265420 PMC3266542

[34] Lin B Yin T Wu YI Inoue T Levchenko A. Interplay between chemotaxis and contact inhibition of locomotion determines exploratory cell migration. Nat Commun. 2015;6:6619. 10.1038/ncomms7619 25851023 PMC4391292

[35] Ouzounova M Lee E Piranlioglu R El Andaloussi A Kolhe R Demirci MF Monocytic and granulocytic myeloid derived suppressor cells differentially regulate spatiotemporal tumour plasticity during metastatic cascade. Nat Commun. 2017;8:14979. 10.1038/ncomms14979 28382931 PMC5384228

[36] Sugiyama N Gucciardo E Tatti O Varjosalo M Hyytiainen M Gstaiger M EphA2 cleavage by MT1-MMP triggers single cancer cell invasion via homotypic cell repulsion. J Cell Biol. 2013;201:467–84. 10.1083/jcb.201205176 23629968 PMC3639392

[37] Tasdogan A Faubert B Ramesh V Ubellacker JM Shen B Solmonson A Metabolic heterogeneity confers differences in melanoma metastatic potential. Nature. 2020;577:115–20. 10.1038/s41586-019-1847-2 31853067 PMC6930341

[38] LeBleu VS O’Connell JT Gonzalez Herrera KN Wikman H Pantel K Haigis MC PGC-1α mediates mitochondrial biogenesis and oxidative phosphorylation in cancer cells to promote metastasis. Nat Cell Biol. 2014;16:992–1003, 1–15. 10.1038/ncb3039 25241037 PMC4369153

[39] Padmanaban V Krol I Suhail Y Szczerba BM Aceto N Bader JS E-cadherin is required for metastasis in multiple models of breast cancer. Nature. 2019;573:439–44. 10.1038/s41586-019-1526-3 31485072 PMC7365572

[40] Piskounova E Agathocleous M Murphy MM Hu Z Huddlestun SE Zhao Z Oxidative stress inhibits distant metastasis by human melanoma cells. Nature. 2015;527:186–91. 10.1038/nature15726 26466563 PMC4644103

[41] Hanna RN Cekic C Sag D Tacke R Thomas GD Nowyhed H Patrolling monocytes control tumor metastasis to the lung. Science. 2015;350:985–90. 10.1126/science.aac9407 26494174 PMC4869713

[42] Ubellacker JM Tasdogan A Ramesh V Shen B Mitchell EC Martin-Sandoval MS Lymph protects metastasizing melanoma cells from ferroptosis. Nature. 2020;585:113–8. 10.1038/s41586-020-2623-z 32814895 PMC7484468

[43] Zhao Z Wu MS Zou C Tang Q Lu J Liu D Downregulation of MCT1 inhibits tumor growth, metastasis and enhances chemotherapeutic efficacy in osteosarcoma through regulation of the NF-κB pathway. Cancer Lett. 2014;342:150–8. 10.1016/j.canlet.2013.08.042 24012639

[44] Barrow-McGee R Kishi N Joffre C Ménard L Hervieu A Bakhouche BA Beta 1-integrin-c-Met cooperation reveals an inside-in survival signalling on autophagy-related endomembranes. Nat Commun. 2016;7:11942. 10.1038/ncomms11942 27336951 PMC4931016

[45] Wolf MJ Hoos A Bauer J Boettcher S Knust M Weber A Endothelial CCR2 signaling induced by colon carcinoma cells enables extravasation via the JAK2-Stat5 and p38MAPK pathway. Cancer Cell. 2012;22:91–105. 10.1016/j.ccr.2012.05.023 22789541

[46] Olmeda D Cerezo-Wallis D Riveiro-Falkenbach E Pennacchi PC Contreras-Alcalde M Ibarz N Whole-body imaging of lymphovascular niches identifies pre-metastatic roles of midkine. Nature. 2017;546:676–80. 10.1038/nature22977 28658220 PMC6005659

[47] Png KJ Halberg N Yoshida M Tavazoie SF. A microRNA regulon that mediates endothelial recruitment and metastasis by cancer cells. Nature. 2011;481:190–4. 10.1038/nature10661 22170610

[48] Karnezis T Shayan R Caesar C Roufail S Harris NC Ardipradja K VEGF-D promotes tumor metastasis by regulating prostaglandins produced by the collecting lymphatic endothelium. Cancer Cell. 2012;21:181–95. 10.1016/j.ccr.2011.12.026 22340592

[49] Lee E Fertig EJ Jin K Sukumar S Pandey NB Popel AS. Breast cancer cells condition lymphatic endothelial cells within pre-metastatic niches to promote metastasis. Nat Commun. 2014;5:4715. 10.1038/ncomms5715 25178650 PMC4351998

[50] Chen XJ Wei WF Wang ZC Wang N Guo CH Zhou CF A novel lymphatic pattern promotes metastasis of cervical cancer in a hypoxic tumour-associated macrophage-dependent manner. Forthcoming 2021. 10.1007/s10456-020-09766-2 PMC8292274 33484377

[51] Labelle M Begum S Hynes RO. Direct signaling between platelets and cancer cells induces an epithelial-mesenchymal-like transition and promotes metastasis. Cancer Cell. 2011;20:576–90. 10.1016/j.ccr.2011.09.009 22094253 PMC3487108

[52] Papa AL Jiang A Korin N Chen MB Langan ET Waterhouse A Platelet decoys inhibit thrombosis and prevent metastatic tumor formation in preclinical models. Sci Transl Med. 2019;11:eaau5898. 10.1126/scitranslmed.aau5898 30760580

[53] Zheng X Liu J Li X Tian R Shang K Dong X Angiogenesis is promoted by exosomal DPP4 derived from 5-fluorouracil-resistant colon cancer cells. Cancer Lett. 2021;497:190–201. 10.1016/j.canlet.2020.10.009 33039561

[54] Schwartz H Blacher E Amer M Livneh N Abramovitz L Klein A Incipient melanoma brain metastases instigate astrogliosis and neuroinflammation. Cancer Res. 2016;76:4359–71. 10.1158/0008-5472.CAN-16-0485 27261506

[55] Strilic B Yang L Albarrán-Juárez J Wachsmuth L Han K Müller UC Tumour-cell-induced endothelial cell necroptosis via death receptor 6 promotes metastasis. Nature. 2016;536:215–8. 10.1038/nature19076 27487218

[56] Wieland E Rodriguez-Vita J Liebler SS Mogler C Moll I Herberich SE Endothelial Notch1 activity facilitates metastasis. Cancer Cell. 2017;31:355–67. 10.1016/j.ccell.2017.01.007 28238683

[57] Connor Y Tekleab S Nandakumar S Walls C Tekleab Y Husain A Physical nanoscale conduit-mediated communication between tumour cells and the endothelium modulates endothelial phenotype. Nat Commun. 2015;6:8671. 10.1038/ncomms9671 26669454 PMC4697439

[58] Wagenblast E Soto M Gutiérrez-Ángel S Hartl CA Gable AL Maceli AR A model of breast cancer heterogeneity reveals vascular mimicry as a driver of metastasis. Nature. 2015;520:358–62. 10.1038/nature14403 25855289 PMC4634366

[59] Lugassy C Kleinman HK Vermeulen PB Barnhill RL. Angiotropism, pericytic mimicry and extravascular migratory metastasis: an embryogenesis-derived program of tumor spread. Angiogenesis. 2020;23:27–41. 10.1007/s10456-019-09695-9 31720876

[60] Aguirre-Ghiso JA. Models, mechanisms and clinical evidence for cancer dormancy. Nat Rev Cancer. 2007;7:834–46. 10.1038/nrc2256 17957189 PMC2519109

[61] Bartosh TJ Ullah M Zeitouni S Beaver J Prockop DJ. Cancer cells enter dormancy after cannibalizing mesenchymal stem/stromal cells (MSCs). Proc Natl Acad Sci U S A. 2016;113:E6447–56. 10.1073/pnas.1612290113 27698134 PMC5081643

[62] Guldner IH Wang Q Yang L Golomb SM Zhao Z Lopez JA CNS-native myeloid cells drive immune suppression in the brain metastatic niche through cxcl10. Cell. 2020;183:1234–48.e25. 10.1016/j.cell.2020.09.064 33113353 PMC7704908

[63] Wu Z Wei D Gao W Xu Y Hu Z Ma Z TPO-induced metabolic reprogramming drives liver metastasis of colorectal cancer CD110+ tumor-initiating cells. Cell Stem Cell. 2015;17:47–59. 10.1016/j.stem.2015.05.016 26140605

[64] Clever D Roychoudhuri R Constantinides MG Askenase MH Sukumar M Klebanoff CA Oxygen sensing by T cells establishes an immunologically tolerant metastatic niche. Cell. 2016;166:1117–31.e14. 10.1016/j.cell.2016.07.032 27565342 PMC5548538

[65] Howe EN Burnette MD Justice ME Schnepp PM Hedrick V Clancy JW Rab11b-mediated integrin recycling promotes brain metastatic adaptation and outgrowth. Nat Commun. 2020;11:3017. 10.1038/s41467-020-16832-2 32541798 PMC7295786

[66] Ramel D Wang X Laflamme C Montell DJ Emery G. Rab11 regulates cell-cell communication during collective cell movements. Nat Cell Biol. 2013;15:317–24. 10.1038/ncb2681 23376974 PMC4006229

[67] Loo JM Scherl A Nguyen A Man FY Weinberg E Zeng Z Extracellular metabolic energetics can promote cancer progression. Cell. 2015;160:393–406. 10.1016/j.cell.2014.12.018 25601461 PMC4312495

[68] Zhang L Zhang S Yao J Lowery FJ Zhang Q Huang WC Microenvironment-induced PTEN loss by exosomal microRNA primes brain metastasis outgrowth. Nature. 2015;527:100–4. 10.1038/nature15376 26479035 PMC4819404

[69] Hugo H Ackland ML Blick T Lawrence MG Clements JA Williams ED Epithelial--mesenchymal and mesenchymal--epithelial transitions in carcinoma progression. J Cell Physiol. 2007;213:374–83. 10.1002/jcp.21223 17680632

[70] Del Pozo Martin Y Park D Ramachandran A Ombrato L Calvo F Chakravarty P Mesenchymal cancer cell-stroma crosstalk promotes niche aactivation, epithelial reversion, and metastatic colonization. Cell Rep. 2015;13:2456–69. 10.1016/j.celrep.2015.11.025 26670048 PMC4695340

[71] Jiao S Subudhi SK Aparicio A Ge Z Guan B Miura Y Differences in tumor microenvironment dictate T helper lineage polarization and response to immune checkpoint therapy. Cell. 2019;179:1177–90 e13. 10.1016/j.cell.2019.10.029 31730856

[72] Houg DS Bijlsma MF. The hepatic pre-metastatic niche in pancreatic ductal adenocarcinoma. Molecular Cancer. 2018;17:95. 10.1186/s12943-018-0842-9 29903049 PMC6003100

[73] Liu Y Gu Y Han Y Zhang Q Jiang Z Zhang X Tumor exosomal RNAs promote lung pre-metastatic niche formation by activating alveolar epithelial TLR3 to recruit neutrophils. Cancer Cell. 2016;30:243–56. 10.1016/j.ccell.2016.06.021 27505671

[74] Barkan D Green JE Chambers AF. Extracellular matrix: a gatekeeper in the transition from dormancy to metastatic growth. Eur J Cancer. 2010;46:1181–8. 10.1016/j.ejca.2010.02.027 20304630 PMC2856784

[75] Cox TR Rumney RMH Schoof EM Perryman L Høye AM Agrawal A The hypoxic cancer secretome induces pre-metastatic bone lesions through lysyl oxidase. Nature. 2015;522:106–10. 10.1038/nature14492 26017313 PMC4961239

[76] Hoshino A Costa-Silva B Shen TL Rodrigues G Hashimoto A Tesic Mark M Tumour exosome integrins determine organotropic metastasis. Nature. 2015;527:329–35. 10.1038/nature15756 26524530 PMC4788391

[77] Headley MB Bins A Nip A Roberts EW Looney MR Gerard A Visualization of immediate immune responses to pioneer metastatic cells in the lung. Nature. 2016;531:513–7. 10.1038/nature16985 26982733 PMC4892380

[78] Cai Z Li CF Han F Liu C Zhang A Hsu CC Phosphorylation of PDHA by AMPK drives TCA cycle to promote cancer metastasis. Mol Cell. 2020;80:263–78.e7. 10.1016/j.molcel.2020.09.018 33022274 PMC7534735

[79] Bu P Chen KY Xiang K Johnson C Crown SB Rakhilin N Aldolase B-mediated fructose metabolism drives metabolic reprogramming of colon cancer liver metastasis. Cell Metab. 2018;27:1249–62.e4. 10.1016/j.cmet.2018.04.003 29706565 PMC5990465

[80] Kim J Kang J Kang YL Woo J Kim Y Huh J Ketohexokinase-A acts as a nuclear protein kinase that mediates fructose-induced metastasis in breast cancer. Nat commun. 2020;11:5436. 10.1038/s41467-020-19263-1 33116123 PMC7595112

[81] Cui C Yang J Li X Liu D Fu L Wang X. Functions and mechanisms of circular RNAs in cancer radiotherapy and chemotherapy resistance. Mol Cancer. 2020;19:58–73. 10.1186/s12943-020-01180-y 32171304 PMC7071709

[82] Hao J. The role of acidic microenvironment in the tumor aggressive phenotypes and the treatment. Tradit Med Res. 2020;5:4–6. 10.12032/TMR20191231153

[83] Qian BZ Pollard JW. Macrophage diversity enhances tumor progression and metastasis. Cell. 2010;141:39–51. 10.1016/j.cell.2010.03.014 20371344 PMC4994190

[84] Nejman D Livyatan I Fuks G Gavert N Zwang Y Geller LT The human tumor microbiome is composed of tumor type-specific intracellular bacteria. Science. 2020;368:973–80. 10.1126/science.aay9189 32467386 PMC7757858

[85] Puram SV Tirosh I Parikh AS Patel AP Yizhak K Gillespie S Single-cell transcriptomic analysis of primary and metastatic tumor ecosystems in head and neck cancer. Cell. 2017;171:1611–24.e24. 10.1016/j.cell.2017.10.044 29198524 PMC5878932

[86] Astin JW Batson J Kadir S Charlet J Persad RA Gillatt D Competition amongst Eph receptors regulates contact inhibition of locomotion and invasiveness in prostate cancer cells. Nat Cell Biol. 2010;12:1194–204. 10.1038/ncb2122 21076414

[87] Ershaid N Sharon Y Doron H Raz Y Shani O Cohen N NLRP3 inflammasome in fibroblasts links tissue damage with inflammation in breast cancer progression and metastasis. Nat Commun. 2019;10:4375. 10.1038/s41467-019-12370-8 31558756 PMC6763472

[88] Wang X Song X Cheng G Zhang J Dong L Bai J The regulatory mechanism and biological significance of mitochondrial calcium uniporter in the migration, invasion, angiogenesis and growth of gastric cancer. Onco Targets Ther. 2020;13:11781–94. 10.2147/OTT.S262049 33235465 PMC7680189

[89] Shang S Ji X Zhang L Chen J Li C Shi R Macrophage ABHD5 suppresses NFκB-dependent matrix metalloproteinase expression and cancer metastasis. Cancer Res. 2019;79:5513–26. 10.1158/0008-5472.CAN-19-1059 31439546

[90] Srivastava K Hu J Korn C Savant S Teichert M Kapel SS Postsurgical adjuvant tumor therapy by combining anti-angiopoietin-2 and metronomic chemotherapy limits metastatic growth. Cancer Cell. 2014;26:880–95. 10.1016/j.ccell.2014.11.005 25490450

[91] Fan QM Jing YY Yu GF Kou XR Ye F Gao L Tumor-associated macrophages promote cancer stem cell-like properties via transforming growth factor-beta1-induced epithelial-mesenchymal transition in hepatocellular carcinoma. Cancer Lett. 2014;352:160–8. 10.1016/j.canlet.2014.05.008 24892648

[92] Yang Y Andersson P Hosaka K Zhang Y Cao R Iwamoto H The PDGF-BB-SOX7 axis-modulated IL-33 in pericytes and stromal cells promotes metastasis through tumour-associated macrophages. Nat Commun. 2016;7:11385. 10.1038/ncomms11385 27150562 PMC4859070

[93] Karnoub AE Dash AB Vo AP Sullivan A Brooks MW Bell GW Mesenchymal stem cells within tumour stroma promote breast cancer metastasis. Nature. 2007;449:557–63. 10.1038/nature06188 17914389

[94] Chen YC Gonzalez ME Burman B Zhao X Anwar T Tran M Mesenchymal stem/stromal cell engulfment reveals metastatic advantage in breast cancer. Cell Rep. 2019;27:3916–26.e5. 10.1016/j.celrep.2019.05.084 31242423 PMC6657699

[95] Jung Y Kim JK Shiozawa Y Wang J Mishra A Joseph J Recruitment of mesenchymal stem cells into prostate tumours promotes metastasis. Nat Commun. 2013;4:1795. 10.1038/ncomms2766 23653207 PMC3649763

[96] Gast CE Silk AD Zarour L Riegler L Burkhart JG Gustafson KT Cell fusion potentiates tumor heterogeneity and reveals circulating hybrid cells that correlate with stage and survival. Sci Adv. 2018;4:eaat7828. 10.1126/sciadv.aat7828 30214939 PMC6135550

[97] Zomer A Maynard C Verweij FJ Kamermans A Schäfer R Beerling E *In vivo* imaging reveals extracellular vesicle-mediated phenocopying of metastatic behavior. Cell. 2015;161:1046–57. 10.1016/j.cell.2015.04.042 26000481 PMC4448148

[98] Neelakantan D Zhou H Oliphant MUJ Zhang X Simon LM Henke DM EMT cells increase breast cancer metastasis via paracrine GLI activation in neighbouring tumour cells. Nat Commun. 2017;8:15773. 10.1038/ncomms15773 28604738 PMC5472791

[99] Kuriyama S Theveneau E Benedetto A Parsons M Tanaka M Charras G *In vivo* collective cell migration requires an LPAR2-dependent increase in tissue fluidity. J Cell Biol. 2014;206:113–27. 10.1083/jcb.201402093 25002680 PMC4085712

[100] Cheung KJ Padmanaban V Silvestri V Schipper K Cohen JD Fairchild AN Polyclonal breast cancer metastases arise from collective dissemination of keratin 14-expressing tumor cell clusters. Proc Natl Acad Sci U S A. 2016;113:E854–63. 10.1073/pnas.1508541113 26831077 PMC4763783

[101] Au SH Storey BD Moore JC Tang Q Chen YL Javaid S Clusters of circulating tumor cells traverse capillary-sized vessels. Proc Natl Acad Sci U S A. 2016;113:4947–52. 10.1073/pnas.1524448113 27091969 PMC4983862

[102] Tuguzbaeva G Yue E Chen X He L Li X Ju J PEP06 polypeptide 30 is a novel cluster-dissociating agent inhibiting αv integrin/FAK/Src signaling in oral squamous cell carcinoma cells. Acta Pharm Sin B. 2019;9:1163–73. 10.1016/j.apsb.2019.10.005 31867162 PMC6900557

[103] Yu S Li L Tian W Nie D Mu W Qiu F PEP06 polypeptide 30 exerts antitumour effect in colorectal carcinoma via inhibiting epithelial-mesenchymal transition. Br J Pharmacol. 2018;175:3111–30. 10.1111/bph.14352 29722931 PMC6031886

[104] Wiel C Le Gal K Ibrahim MX Jahangir CA Kashif M Yao H BACH1 stabilization by antioxidants stimulates lung cancer metastasis. Cell. 2019;178:330–45.e22. 10.1016/j.cell.2019.06.005 31257027

[105] Lugini L Matarrese P Tinari A Lozupone F Federici C Iessi E Cannibalism of live lymphocytes by human metastatic but not primary melanoma cells. Cancer Res. 2006;66:3629–38. 10.1158/0008-5472.CAN-05-3204 16585188

[106] Pascual G Avgustinova A Mejetta S Martín M Castellanos A Attolini CS Targeting metastasis-initiating cells through the fatty acid receptor CD36. Nature. 2017;541:41–5. 10.1038/nature20791 27974793

[107] Lee CK Jeong SH Jang C Bae H Kim YH Park I Tumor metastasis to lymph nodes requires YAP-dependent metabolic adaptation. Science. 2019;363:644–9. 10.1126/science.aav0173 30733421

[108] Bhattacharya B Mohd Omar MF Soong R. The Warburg effect and drug resistance. Br J Pharmacol. 2016;173:970–9. 10.1111/bph.13422 26750865 PMC4793921

[109] Cordero A Kanojia D Miska J Panek WK Xiao A Han Y FABP7 is a key metabolic regulator in HER^2+^ breast cancer brain metastasis. Oncogene. 2019;38:6445–60. 10.1038/s41388-019-0893-4 31324889 PMC6742563

[110] Gu L Larson Casey JL Andrabi SA Lee JH Meza-Perez S Randall TD Mitochondrial calcium uniporter regulates PGC-1α expression to mediate metabolic reprogramming in pulmonary fibrosis. Redox Biol. 2019;26:101307. 10.1016/j.redox.2019.101307 31473487 PMC6831865

[111] Deng M Gui X Kim J Xie L Chen W Li Z LILRB4 signalling in leukaemia cells mediates T cell suppression and tumour infiltration. Nature. 2018;562:605–9. 10.1038/s41586-018-0615-z 30333625 PMC6296374

[112] Adiga D Radhakrishnan R Chakrabarty S Kumar P Kabekkodu SP. The role of calcium signaling in regulation of epithelial-mesenchymal transition. Cells Tissues Organs. Forthcoming 2021. 10.1159/000512277 33316804

[113] Leverrier-Penna S Destaing O Penna A. Insights and perspectives on calcium channel functions in the cockpit of cancerous space invaders. Cell Calcium. 2020;90:102251. 10.1016/j.ceca.2020.102251 32683175

[114] Bauer KS Cude KJ Dixon SC Kruger EA Figg WD. Carboxyamido-triazole inhibits angiogenesis by blocking the calcium-mediated nitric-oxide synthase-vascular endothelial growth factor pathway. J Pharmacol Exp Ther. 2000;292:31–7. 10604929

[115] Almquist M Anagnostaki L Bondeson L Bondeson AG Borgquist S Landberg G Serum calcium and tumour aggressiveness in breast cancer: a prospective study of 7847 women. Eur J Cancer Prev. 2009;18:354–60. 10.1097/CEJ.0b013e32832c386f 19593149

[116] Li XC Dong YY Cheng Y Zhou JY Yang X Shen BQ Increased serum calcium level promotes the risk of lymph node metastasis in endometrial cancer. Cancer Manag Res. 2020;12:5023–30. 10.2147/CMAR.S253914 32612389 PMC7323808

[117] Huang SY Chen Y Tan XR Gong S Yang XJ He QM Serum calcium levels before antitumour therapy predict clinical outcomes in patients with nasopharyngeal carcinoma. Onco Targets Ther. 2020;13:13111–9. 10.2147/OTT.S275613 33380801 PMC7767708

[118] Tsuji Y Nakamori S Ariyoshi H Sakon M Aono Y Ueda A Cancer cell contact causes oscillatory and polarized rise of cytoplasmic ionized calcium concentration in human umbilical vein endothelial cells. Int J Oncol. 2002;21:541–6. 12168097

[119] Wu D Ma X Lin F. DC electric fields direct breast cancer cell migration, induce EGFR polarization, and increase the intracellular level of calcium ions. Cell Biochem Biophys. 2013;67:1115–25. 10.1007/s12013-013-9615-7 23657921

[120] Pon CK Lane JR Sloan EK Halls ML. The β_2_-adrenoceptor activates a positive cAMP-calcium feedforward loop to drive breast cancer cell invasion. FASEB J. 2016;30:1144–54. 10.1096/fj.15-277798 26578688 PMC4750412

[121] Hwang YP Jeong HG. Metformin blocks migration and invasion of tumour cells by inhibition of matrix metalloproteinase-9 activation through a calcium and protein kinase Calpha-dependent pathway: phorbol-12-myristate-13-acetate-induced/extracellular signal-regulated kinase/activator protein-1. Br J Pharmacol. 2010;160:1195–211. 10.1111/j.1476-5381.2010.00762.x 20590612 PMC2936028

[122] Timar J Chopra H Rong X Hatfield JS Fligiel SE Onoda JM Calcium channel blocker treatment of tumor cells induces alterations in the cytoskeleton, mobility of the integrin alpha IIb beta 3 and tumor-cell-induced platelet aggregation. J Cancer Res Clin Oncol. 1992;118:425–34. 10.1007/BF01629425 1377695 PMC12200261

[123] Wang H Gao X Yang JJ Liu ZR. Interaction between p68 RNA helicase and Ca^2+^-calmodulin promotes cell migration and metastasis. Nat Commun. 2013;4:1354. 10.1038/ncomms2345 23322042 PMC3552336

[124] Villalobo A Berchtold MW. The role of calmodulin in tumor cell migration, invasiveness, and metastasis. Int J Mol Sci. 2020;21:765. 10.3390/ijms21030765 PMC7037201 31991573

[125] Liu Z Han G Cao Y Wang Y Gong H. Calcium/calmodulin-dependent protein kinase II enhances metastasis of human gastric cancer by upregulating nuclear factor-κB and Akt-mediated matrix metalloproteinase-9 production. Mol Med Rep. 2014;10:2459–64. 10.3892/mmr.2014.2525 25174603

[126] Sheng W Wang G Tang J Shi X Cao R Sun J Calreticulin promotes EMT in pancreatic cancer via mediating Ca^2+^ dependent acute and chronic endoplasmic reticulum stress. J Exp Clin Cancer Res. 2020;39:209. 10.1186/s13046-020-01702-y 33028359 PMC7542892

[127] Grosshans HK Fischer TT Steinle JA Brill AL Ehrlich BE. Neuronal Calcium Sensor 1 is up-regulated in response to stress to promote cell survival and motility in cancer cells. Mol Oncol. 2020;14:1134–51. 10.1002/1878-0261.12678 32239615 PMC7266285

[128] Xie R Xu J Xiao Y Wu J Wan H Tang B Calcium promotes human gastric cancer via a novel coupling of calcium-sensing receptor and TRPV4 channel. Cancer Res. 2017;77:6499–512. 10.1158/0008-5472.CAN-17-0360 28951460

[129] Chan KT Bennin DA Huttenlocher A. Regulation of adhesion dynamics by calpain-mediated proteolysis of focal adhesion kinase (FAK). J Biol Chem. 2010;285:11418–26. 10.1074/jbc.M109.090746 20150423 PMC2857020

[130] Zheng JC Chang KJ Jin YX Zhao XW Li B Yang MH. Arsenic trioxide inhibits the metastasis of small cell lung cancer by blocking calcineurin-nuclear factor of activated T cells (NFAT) signaling. Med Sci Monit. 2019;25:2228–37. 10.12659/MSM.913091 30913205 PMC6446656

[131] Zhou X Liu Y You J Zhang H Zhang X Ye L. Myosin light-chain kinase contributes to the proliferation and migration of breast cancer cells through cross-talk with activated ERK1/2. Cancer Letters. 2008;270:312–27. 10.1016/j.canlet.2008.05.028 18710790

[132] Luu HH Zhou L Haydon RC Deyrup AT Montag AG Huo D Increased expression of S100A6 is associated with decreased metastasis and inhibition of cell migration and anchorage independent growth in human osteosarcoma. Cancer Lett. 2005;229:135–48. 10.1016/j.canlet.2005.02.015 16157226

[133] Li T Yi L Hai L Ma H Tao Z Zhang C The interactome and spatial redistribution feature of Ca^2+^ receptor protein calmodulin reveals a novel role in invadopodia-mediated invasion. Cell Death Dis. 2018;9:292. 10.1038/s41419-017-0253-7 29463791 PMC5833463

[134] Joeckel E Haber T Prawitt D Junker K Hampel C Thüroff JW High calcium concentration in bones promotes bone metastasis in renal cell carcinomas expressing calcium-sensing receptor. Mol Cancer. 2014;13:42. 10.1186/1476-4598-13-42 24576174 PMC3945739

[135] Apasu JE Schuette D LaRanger R Steinle JA Nguyen LD Grosshans HK Neuronal calcium sensor 1 (NCS1) promotes motility and metastatic spread of breast cancer cells *in vitro* and *in vivo*. FASEB J. 2019;33:4802–13. 10.1096/fj.201802004R 30592625 PMC6436647

[136] Moriyama-Kita M Endo Y Yonemura Y Heizmann CW Miyamori H Sato H S100A4 regulates E-cadherin expression in oral squamous cell carcinoma. Cancer Lett. 2005;230:211–8. 10.1016/j.canlet.2004.12.046 16297707

[137] Jiao J González Á Stevenson HL Gagea M Sugimoto H Kalluri R Depletion of S100A4^+^ stromal cells does not prevent HCC development but reduces the stem cell-like phenotype of the tumors. Exp Mol Med. 2018;50:e422. 10.1038/emm.2017.175 29303514 PMC5992984

[138] Wei R Zhu WW Yu GY Wang X Gao C Zhou X S100 calcium-binding protein A9 from tumor-associated macrophage enhances cancer stem cell-like properties of hepatocellular carcinoma. Int J Cancer. 2020;148:1233–44. 10.1002/ijc.33371 33205453

[139] Duan L Wu R Zhang X Wang D You Y Zhang Y HBx-induced S100A9 in NF-κB dependent manner promotes growth and metastasis of hepatocellular carcinoma cells. Cell Death Dis. 2018;9:629. 10.1038/s41419-018-0512-2 29795379 PMC5967311

[140] Li N Liu L Li G Xia M Du C Zheng Z. The role of BKCa in endometrial cancer HEC-1-B cell proliferation and migration. Gene. 2018;655:42–7. 10.1016/j.gene.2018.02.055 29477869

[141] Hu J Yuan X Ko MK Yin D Sacapano MR Wang X Calcium-activated potassium channels mediated blood-brain tumor barrier opening in a rat metastatic brain tumor model. Mol Cancer. 2007;6:22. 10.1186/1476-4598-6-22 17359538 PMC1831484

[142] Potier M Chantome A Joulin V Girault A Roger S Besson P The SK3/K(Ca)2.3 potassium channel is a new cellular target for edelfosine. Br J Pharmacol. 2011;162:464–79. 10.1111/j.1476-5381.2010.01044.x 20955368 PMC3031066

[143] Wu L Lin W Liao Q Wang H Lin C Tang L Calcium channel blocker nifedipine suppresses colorectal cancer progression and immune escape by preventing NFAT2 nuclear translocation. Cell Rep. 2020;33:108327. 10.1016/j.celrep.2020.108327 33113363

[144] Hao J Bao X Jin B Wang X Mao Z Li X Ca^2+^ channel subunit α 1D promotes proliferation and migration of endometrial cancer cells mediated by 17β-estradiol via the G protein-coupled estrogen receptor. FASEB J. 2015;29:2883–93. 10.1096/fj.14-265603 25805831

[145] Jacquemet G Baghirov H Georgiadou M Sihto H Peuhu E Cettour-Janet P L-type calcium channels regulate filopodia stability and cancer cell invasion downstream of integrin signalling. Nat Commun. 2016;7:13297. 10.1038/ncomms13297 27910855 PMC5146291

[146] Jin YM Ye Y Bao WQ Tong Y Ni SB Liu JP CACNA1B facilitates breast cancer cell growth and migration by regulating cyclin D1 and EMT: the implication of CACNA1B in breast cancer. J Recept Signal Transduct. Forthcoming 2021. 10.1080/10799893.2020.1837871 33100116

[147] Zhang Y Zhang J Jiang D Zhang D Qian Z Liu C Inhibition of T-type Ca^2+^ channels by endostatin attenuates human glioblastoma cell proliferation and migration. Br J Pharmacol. 2012;166:1247–60. 10.1111/j.1476-5381.2012.01852.x 22233416 PMC3417444

[148] Maiques O Barceló C Panosa A Pijuan J Orgaz JL Rodriguez-Hernandez I T-type calcium channels drive migration/invasion in BRAFV600E melanoma cells through Snail1. Pigment Cell Melanoma Res. 2018;31:484–95. 10.1111/pcmr.12690 29385656

[149] Leung CS Yeung TL Yip KP Pradeep S Balasubramanian L Liu J Calcium-dependent FAK/CREB/TNNC1 signalling mediates the effect of stromal MFAP5 on ovarian cancer metastatic potential. Nat Commun. 2014;5:5092. 10.1038/ncomms6092 25277212 PMC4185407

[150] Zhu M Chen L Zhao P Zhou H Zhang C Yu S Store-operated Ca^2+^ entry regulates glioma cell migration and invasion via modulation of Pyk2 phosphorylation. J Exp Clin Cancer Res. 2014;33:98. 10.1186/s13046-014-0098-1 25433371 PMC4258251

[151] Goswamee P Pounardjian T Giovannucci DR. Arachidonic acid-induced Ca^2+^ entry and migration in a neuroendocrine cancer cell line. Cancer Cell Int. 2018;18:30. 10.1186/s12935-018-0529-8 29507531 PMC5834873

[152] Casas-Rua V Tomas-Martin P Lopez-Guerrero AM Alvarez IS Pozo-Guisado E Martin-Romero FJ. STIM1 phosphorylation triggered by epidermal growth factor mediates cell migration. Biochim Biophys Acta. 2015;1853:233–43. 10.1016/j.bbamcr.2014.10.027 25447552

[153] Xia J Wang H Huang H Sun L Dong S Huang N Elevated Orai1 and STIM1 expressions upregulate MACC1 expression to promote tumor cell proliferation, metabolism, migration, and invasion in human gastric cancer. Cancer Lett. 2016;381:31–40. 10.1016/j.canlet.2016.07.014 27431311

[154] Wu S Chen M Huang J Zhang F Lv Z Jia Y ORAI2 promotes gastric cancer tumorigenicity and metastasis through PI3K/Akt signaling and MAPK-dependent focal adhesion disassembly. Cancer Res. 2020;81:986–1000. 10.1158/0008-5472.CAN-20-0049 33310726

[155] Sun J Lu F He H Shen J Messina J Mathew R STIM1- and Orai1-mediated Ca^2+^ oscillation orchestrates invadopodium formation and melanoma invasion. J Cell Biol. 2014;207:535–48. 10.1083/jcb.201407082 25404747 PMC4242838

[156] Koslowski M Türeci O Huber C Sahin U. Selective activation of tumor growth-promoting Ca^2+^ channel MS4A12 in colon cancer by caudal type homeobox transcription factor CDX2. Mol Cancer. 2009;8:77. 10.1186/1476-4598-8-77 19781065 PMC2759907

[157] Zhang LY Zhang YQ Zeng YZ Zhu JL Chen H Wei XL TRPC1 inhibits the proliferation and migration of estrogen receptor-positive Breast cancer and gives a better prognosis by inhibiting the PI3K/AKT pathway. Breast Cancer Res Treat. 2020;182:21–33. 10.1007/s10549-020-05673-8 32415497

[158] Oda K Umemura M Nakakaji R Tanaka R Sato I Nagasako A Transient receptor potential cation 3 channel regulates melanoma proliferation and migration. J Physiol Sci. 2017;67:497–505. 10.1007/s12576-016-0480-1 27613608 PMC10717062

[159] Wei WC Huang WC Lin YP Becker EBE Ansorge O Flockerzi V Functional expression of calcium-permeable canonical transient receptor potential 4-containing channels promotes migration of medulloblastoma cells. J Physiol. 2017;595:5525–44. 10.1113/JP274659 28627017 PMC5556167

[160] Chen Z Zhu Y Dong Y Zhang P Han X Jin J Overexpression of TrpC5 promotes tumor metastasis via the HIF-1alpha-Twist signaling pathway in colon cancer. Clin Sci (Lond). 2017;131:2439–50. 10.1042/CS20171069 28864720

[161] Jardin I Diez-Bello R Lopez JJ Redondo PC Salido GM Smani T TRPC6 channels are required for proliferation, migration and invasion of breast cancer cell lines by modulation of orai1 and orai3 surface exposure. Cancers (Basel). 2018;10:331. 10.3390/cancers10090331 PMC6162527 30223530

[162] Kim JH Hwang KH Eom M Kim M Park EY Jeong Y WNK1 promotes renal tumor progression by activating TRPC6-NFAT pathway. FASEB J. 2019;33:8588–99. 10.1096/fj.201802019RR 31022353

[163] Song Y Liu G Liu S Chen R Wang N Liu Z Helicobacter pylori upregulates TRPC6 via Wnt/beta-catenin signaling to promote gastric cancer migration and invasion. Onco Targets Ther. 2019;12:5269–79 10.2147/OTT.S201025 31308697 PMC6613196

[164] Almasi S Sterea AM Fernando W Clements DR Marcato P Hoskin DW TRPM2 ion channel promotes gastric cancer migration, invasion and tumor growth through the AKT signaling pathway. Sci Rep. 2019;9:4182. 10.1038/s41598-019-40330-1 30862883 PMC6414629

[165] Li W Yang FQ Sun CM Huang JH Zhang HM Li X circPRRC2A promotes angiogenesis and metastasis through epithelial-mesenchymal transition and upregulates TRPM3 in renal cell carcinoma. Theranostics. 2020;10:4395–409. 10.7150/thno.43239 32292503 PMC7150475

[166] Gao Y Liao P. TRPM4 channel and cancer. Cancer Lett. 2019;454:66–9. 10.1016/j.canlet.2019.04.012 30980865

[167] Sagredo AI Sagredo EA Pola V Echeverría C Andaur R Michea L TRPM4 channel is involved in regulating epithelial to mesenchymal transition, migration, and invasion of prostate cancer cell lines. J Cell Physiol. 2019;234:2037–50. 10.1002/jcp.27371 30343491

[168] Maeda T Suzuki A Koga K Miyamoto C Maehata Y Ozawa S TRPM5 mediates acidic extracellular pH signaling and TRPM5 inhibition reduces spontaneous metastasis in mouse B16-BL6 melanoma cells. Oncotarget. 2017;8:78312–26. 10.18632/oncotarget.20826 29108231 PMC5667964

[169] Meng X Cai C Wu J Cai S Ye C Chen H TRPM7 mediates breast cancer cell migration and invasion through the MAPK pathway. Cancer Lett. 2013;333:96–102. 10.1016/j.canlet.2013.01.031 23353055

[170] Chen JP Wang J Luan Y Wang CX Li WH Zhang JB TRPM7 promotes the metastatic process in human nasopharyngeal carcinoma. Cancer Lett. 2015;356:483–90. 10.1016/j.canlet.2014.09.032 25304381

[171] Liu L Wu N Wang Y Zhang X Xia B Tang J TRPM7 promotes the epithelial-mesenchymal transition in ovarian cancer through the calcium-related PI3K/AKT oncogenic signaling. J Exp Clin Cancer Res. 2019;38:106. 10.1186/s13046-019-1061-y 30819230 PMC6396458

[172] Liu JJ Li LZ Xu P. Upregulation of TRPM8 can promote the colon cancer liver metastasis through mediating Akt/GSK-3 signal pathway. Biotechnol Appl Biochem. Forthcoming 2021. 10.1002/bab.2102 33432591

[173] Lyu L Jin X Li Z Liu S Li Y Su R TBBPA regulates calcium-mediated lysosomal exocytosis and thereby promotes invasion and migration in hepatocellular carcinoma. Ecotoxicol Environ Saf. 2020;192:110255. 10.1016/j.ecoenv.2020.110255 32018154

[174] Wu K Shen B Jiang F Xia L Fan T Qin M TRPP2 enhances metastasis by regulating epithelial-mesenchymal transition in laryngeal squamous cell carcinoma. Cell Physiol Biochem. 2016;39:2203–15. 10.1159/000447914 27832627

[175] Gao N Yang F Chen S Wan H Zhao X Dong H. The role of TRPV1 ion channels in the suppression of gastric cancer development. J Exp Clin Cancer Res. 2020;39:206. 10.1186/s13046-020-01707-7 33008449 PMC7531167

[176] Siveen KS Nizamuddin PB Uddin S Al-Thani M Frenneaux MP Janahi IA TRPV2: a cancer biomarker and potential therapeutic target. Dis Markers. 2020;2020:8892312. 10.1155/2020/8892312 33376561 PMC7746447

[177] Li X Cheng Y Wang Z Zhou J Jia Y He X Calcium and TRPV4 promote metastasis by regulating cytoskeleton through the RhoA/ROCK1 pathway in endometrial cancer. Cell Death Dis. 2020;11:1009. 10.1038/s41419-020-03181-7 33230171 PMC7683721

[178] Lee WH Choong LY Jin TH Mon NN Chong S Liew CS TRPV4 plays a role in breast cancer cell migration via Ca^2+^-dependent activation of AKT and downregulation of E-cadherin cell cortex protein. Oncogenesis. 2017;6:e338. 10.1038/oncsis.2017.39 28530703 PMC5523072

[179] Cappelli HC Kanugula AK Adapala RK Amin V Sharma P Midha P Mechanosensitive TRPV4 channels stabilize VE-cadherin junctions to regulate tumor vascular integrity and metastasis. Cancer Lett. 2019;442:15–20. 10.1016/j.canlet.2018.07.042 30401632 PMC6924277

[180] Chen Y Liu X Zhang F Liao S He X Zhuo D Vitamin D receptor suppresses proliferation and metastasis in renal cell carcinoma cell lines via regulating the expression of the epithelial Ca^2+^ channel TRPV5. PLoS One. 2018;13:e0195844. 10.1371/journal.pone.0195844 29659618 PMC5901920

[181] Raphaël M Lehen’kyi V Vandenberghe M Beck B Khalimonchyk S Vanden Abeele F TRPV6 calcium channel translocates to the plasma membrane via Orai1-mediated mechanism and controls cancer cell survival. Proc Natl Acad Sci U S A. 2014;111:E3870-9. 10.1073/pnas.1413409111 25172921 PMC4169956

[182] Wang T Li N Jin L Qi X Zhang C Hua D. The calcium pump PMCA4 prevents epithelial-mesenchymal transition by inhibiting NFATc1-ZEB1 pathway in gastric cancer. Biochim Biophys Acta Mol Cell Res. 2020;1867:118833. 10.1016/j.bbamcr.2020.118833 32860837

[183] Ryu S McDonnell K Choi H Gao D Hahn M Joshi N Suppression of miRNA-708 by polycomb group promotes metastases by calcium-induced cell migration. Cancer Cell. 2013;23:63–76. 10.1016/j.ccr.2012.11.019 23328481

[184] Chung FY Lin SR Lu CY Yeh CS Chen FM Hsieh JS Sarco/endoplasmic reticulum calcium-ATPase 2 expression as a tumor marker in colorectal cancer. Am J Surg Pathol. 2006;30:969–74. 10.1097/00000478-200608000-00006 16861967

[185] Gou WF Niu ZF Zhao S Takano Y Zheng HC. Aberrant SERCA3 expression during the colorectal adenoma-adenocarcinoma sequence. Oncol Rep. 2014;31:232–40. 10.3892/or.2013.2837 24213720

[186] Brisson L Chadet S Lopez-Charcas O Jelassi B Ternant D Chamouton J P2X7 receptor promotes mouse mammary cancer cell invasiveness and tumour progression, and is a target for anticancer treatment. Cancers (Basel). 2020;12:2342. 10.3390/cancers12092342 PMC7565976 32825056

[187] Zhang WJ Hu CG Luo HL Zhu ZM. Activation of P2X7 receptor promotes the invasion and migration of colon cancer cells via the STAT3 Signaling. Front Cell Dev Biol. 2020;8:586555. 10.3389/fcell.2020.586555 33330466 PMC7732635

[188] Zhang Y Cheng H Li W Wu H Yang Y. Highly-expressed P2X7 receptor promotes growth and metastasis of human HOS/MNNG osteosarcoma cells via PI3K/Akt/GSK3β/β-catenin and mTOR/HIF1α/VEGF signaling. Int J Cancer. 2019;145:1068–82. 10.1002/ijc.32207 30761524 PMC6618011

[189] Chen L He HY Li HM Zheng J Heng WJ You JF ERK1/2 and p38 pathways are required for P2Y receptor-mediated prostate cancer invasion. Cancer Lett. 2004;215:239–47. 10.1016/j.canlet.2004.05.023 15488643

[190] Li WH Qiu Y Zhang HQ Liu Y You JF Tian XX P2Y2 receptor promotes cell invasion and metastasis in prostate cancer cells. Br J Cancer. 2013;109:1666–75. 10.1038/bjc.2013.484 23969730 PMC3776994

[191] Girard M Dagenais Bellefeuille S Eiselt É Brouillette R Placet M Arguin G The P2Y_6_ receptor signals through Gα_q_/Ca^2+^/PKCα and Gα_13_/ROCK pathways to drive the formation of membrane protrusions and dictate cell migration. J Cell Physiol. 2020;235:9676–90. 10.1002/jcp.29779 32420639

[192] Kamiyama M Shirai T Tamura S Suzuki-Inoue K Ehata S Takahashi K ASK1 facilitates tumor metastasis through phosphorylation of an ADP receptor P2Y_12_ in platelets. Cell Death Differ. 2017;24:2066–76. 10.1038/cdd.2017.114 28753204 PMC5686340

[193] Chen L Sun Q Zhou D Song W Yang Q Ju B HINT2 triggers mitochondrial Ca^2+^ influx by regulating the mitochondrial Ca^2+^ uniporter (MCU) complex and enhances gemcitabine apoptotic effect in pancreatic cancer. Cancer Lett. 2017;411:106–16. 10.1016/j.canlet.2017.09.020 28947137

[194] Ren T Zhang H Wang J Zhu J Jin M Wu Y MCU-dependent mitochondrial Ca^2+^ inhibits NAD^+^/SIRT3/SOD2 pathway to promote ROS production and metastasis of HCC cells. Oncogene. 2017;36:5897–909. 10.1038/onc.2017.167 28650465

[195] Zheng X Lu S He Z Huang H Yao Z Miao Y MCU-dependent negative sorting of miR-4488 to extracellular vesicles enhances angiogenesis and promotes breast cancer metastatic colonization. Oncogene. 2020;39:6975–89. 10.1038/s41388-020-01514-6 33067576

[196] Tosatto A Sommaggio R Kummerow C Bentham RB Blacker TS Berecz T The mitochondrial calcium uniporter regulates breast cancer progression via HIF-1α. EMBO Mol Med. 2016;8:569–85. 10.15252/emmm.201606255 27138568 PMC4864890

[197] Jin M Wang J Ji X Cao H Zhu J Chen Y MCUR1 facilitates epithelial-mesenchymal transition and metastasis via the mitochondrial calcium dependent ROS/Nrf2/Notch pathway in hepatocellular carcinoma. J Exp Clin Cancer Res. 2019;38:136. 10.1186/s13046-019-1135-x 30909929 PMC6434841

[198] D’Amore A Hanbashi AA Di Agostino S Palombi F Sacconi A Voruganti A Loss of two-pore channel 2 (TPC2) expression increases the metastatic traits of melanoma cells by a mechanism involving the hippo signalling pathway and store-operated calcium entry. Cancers (Basel). 2020;12:2391. 10.3390/cancers12092391 PMC7564716 32846966

[199] Shibao K Fiedler MJ Nagata J Minagawa N Hirata K Nakayama Y The type III inositol 1,4,5-trisphosphate receptor is associated with aggressiveness of colorectal carcinoma. Cell calcium. 2010;48:315–23. 10.1016/j.ceca.2010.09.005 21075448 PMC3572849

[200] Xu N Zhang D Chen J He G Gao L. Low expression of ryanodine receptor 2 is associated with poor prognosis in thyroid carcinoma. Oncol Lett. 2019;18:3605–12. 10.3892/ol.2019.10732 31516575 PMC6732998

[201] Cui C Chang Y Zhang X Choi S Tran H Penmetsa KV Targeting Orai1-mediated store-operated calcium entry by RP4010 for anti-tumor activity in esophagus squamous cell carcinoma. Cancer Lett. 2018;432:169–79. 10.1016/j.canlet.2018.06.006 29908962 PMC6070391

[202] Yu C Tang W Wang Y Shen Q Wang B Cai C Downregulation of ACE2/Ang-(1-7)/Mas axis promotes breast cancer metastasis by enhancing store-operated calcium entry. Cancer Lett. 2016;376:268–77. 10.1016/j.canlet.2016.04.006 27063099

[203] Li J McKeown L Ojelabi O Stacey M Foster R O’Regan D Nanomolar potency and selectivity of a Ca^2+^ release-activated Ca^2+^ channel inhibitor against store-operated Ca^2+^ entry and migration of vascular smooth muscle cells. Br J Pharmacol. 2011;164:382–93. 10.1111/j.1476-5381.2011.01368.x 21545575 PMC3174418

[204] Hammadi M Chopin V Matifat F Dhennin-Duthille I Chasseraud M Sevestre H Human ether à-gogo K^+^ channel 1 (hEag1) regulates MDA-MB-231 breast cancer cell migration through Orai1-dependent calcium entry. J Cell Physiol. 2012;227:3837–46. 10.1002/jcp.24095 22495877

[205] Zhang X Zhang L Lin B Chai X Li R Liao Y Phospholipid Phosphatase 4 promotes proliferation and tumorigenesis, and activates Ca^2+^-permeable Cationic Channel in lung carcinoma cells. Mol Cancer. 2017;16:147. 10.1186/s12943-017-0717-5 28851360 PMC5576330

[206] Gershkovitz M Caspi Y Fainsod-Levi T Katz B Michaeli J Khawaled S TRPM2 mediates neutrophil killing of disseminated tumor cells. Cancer Res. 2018;78:2680–90. 10.1158/0008-5472.CAN-17-3614 29490946

[207] Kanugula AK Adapala RK Midha P Cappelli HC Meszaros JG Paruchuri S Novel noncanonical regulation of soluble VEGF/VEGFR2 signaling by mechanosensitive ion channel TRPV4. FASEB J. 2019;33:195–203. 10.1096/fj.201800509R 29957061 PMC6355071

[208] Cui C Yang J Fu L Wang M Wang X. Progress in understanding mitochondrial calcium uniporter complex-mediated calcium signalling: a potential target for cancer treatment. Br J Pharmacol. 2019;176:1190–205. 10.1111/bph.14632 30801705 PMC6468257

[209] Gu L Larson-Casey JL Carter AB. Macrophages utilize the mitochondrial calcium uniporter for profibrotic polarization. FASEB J. 2017;31:3072–83. 10.1096/fj.201601371R 28351840 PMC5471522

[210] Fouque A Lepvrier E Debure L Gouriou Y Malleter M Delcroix V The apoptotic members CD95, BclxL, and Bcl-2 cooperate to promote cell migration by inducing Ca^2+^ flux from the endoplasmic reticulum to mitochondria. Cell Death Differ. 2016;23:1702–16. 10.1038/cdd.2016.61 27367565 PMC5041197

[211] Ndiaye D Collado-Hilly M Martin J Prigent S Dufour JF Combettes L Characterization of the effect of the mitochondrial protein Hint2 on intracellular Ca^2+^ dynamics. Biophys J. 2013;105:1268–75. 10.1016/j.bpj.2013.06.048 24010670 PMC3762358

[212] Davis FM Parsonage MT Cabot PJ Parat MO Thompson EW Roberts-Thomson SJ Assessment of gene expression of intracellular calcium channels, pumps and exchangers with epidermal growth factor-induced epithelial-mesenchymal transition in a breast cancer cell line. Cancer Cell Int. 2013;13:76. 10.1186/1475-2867-13-76 23890218 PMC3733826

[213] Kang S Hong J Lee JM Moon HE Jeon B Choi J Trifluoperazine, a well-known antipsychotic, inhibits glioblastoma invasion by binding to calmodulin and disinhibiting calcium release channel IP_3_R. Mol Cancer Ther. 2017;16:217–27. 10.1158/1535-7163.MCT-16-0169-T 28062709

